# Replication Fork Polarity Gradients Revealed by Megabase-Sized U-Shaped Replication Timing Domains in Human Cell Lines

**DOI:** 10.1371/journal.pcbi.1002443

**Published:** 2012-04-05

**Authors:** Antoine Baker, Benjamin Audit, Chun-Long Chen, Benoit Moindrot, Antoine Leleu, Guillaume Guilbaud, Aurélien Rappailles, Cédric Vaillant, Arach Goldar, Fabien Mongelard, Yves d'Aubenton-Carafa, Olivier Hyrien, Claude Thermes, Alain Arneodo

**Affiliations:** 1 Université de Lyon, Lyon, France; 2 Laboratoire Joliot-Curie, CNRS, Ecole Normale Supérieure de Lyon, Lyon, France; 3 Laboratoire de Physique, CNRS, Ecole Normale Supérieure de Lyon, Lyon, France; 4 Centre de Génétique Moléculaire UPR 3404, CNRS, Gif-sur-Yvette, France; 5 Institut de Biologie de l'Ecole Normale Supérieure, CNRS UMR8197, Inserm U1024, Paris, France; 6 Commissariat à l'énergie atomique, iBiTecS, Gif-sur-Yvette, France; 7 Laboratoire de Biologie Moléculaire de la Cellule, CNRS, Ecole Normale Supérieure de Lyon, Lyon, France; University of Washington, United States of America

## Abstract

In higher eukaryotes, replication program specification in different cell types remains to be fully understood. We show for seven human cell lines that about half of the genome is divided in domains that display a characteristic U-shaped replication timing profile with early initiation zones at borders and late replication at centers. Significant overlap is observed between U-domains of different cell lines and also with germline replication domains exhibiting a N-shaped nucleotide compositional skew. From the demonstration that the average fork polarity is directly reflected by both the compositional skew and the derivative of the replication timing profile, we argue that the fact that this derivative displays a N-shape in U-domains sustains the existence of large-scale gradients of replication fork polarity in somatic and germline cells. Analysis of chromatin interaction (Hi-C) and chromatin marker data reveals that U-domains correspond to high-order chromatin structural units. We discuss possible models for replication origin activation within U/N-domains. The compartmentalization of the genome into replication U/N-domains provides new insights on the organization of the replication program in the human genome.

## Author Summary

DNA replication in human cells requires the parallel progression along the genome of thousands of replication machineries. Comprehensive knowledge of genetic inheritance at different development stages relies on elucidating the mechanisms that regulate the location and progression of these machineries throughout the duration of the DNA synthetic phase of the cell cycle. Here, we determine in multiple human cell types the existence of a new type of megabase-sized replication domains across which the average orientation of the replication machinery changes in a linear manner. These domains are revealed in 7 somatic cell types by a U-shaped pattern in the replication timing profiles as well as by N-shaped patterns in the DNA compositional asymmetry profile reflecting the existence of a replication-associated mutational asymmetry in the germline. These domains therefore correspond to a robust mode of replication across cell types and during evolution. Using genome-wide data on the frequency of interaction of distant chromatin segments in two cell lines, we find that these U/N-replication domains remarkably correspond to self-interacting folding units of the chromatin fiber.

## Introduction

**Figure 1:**
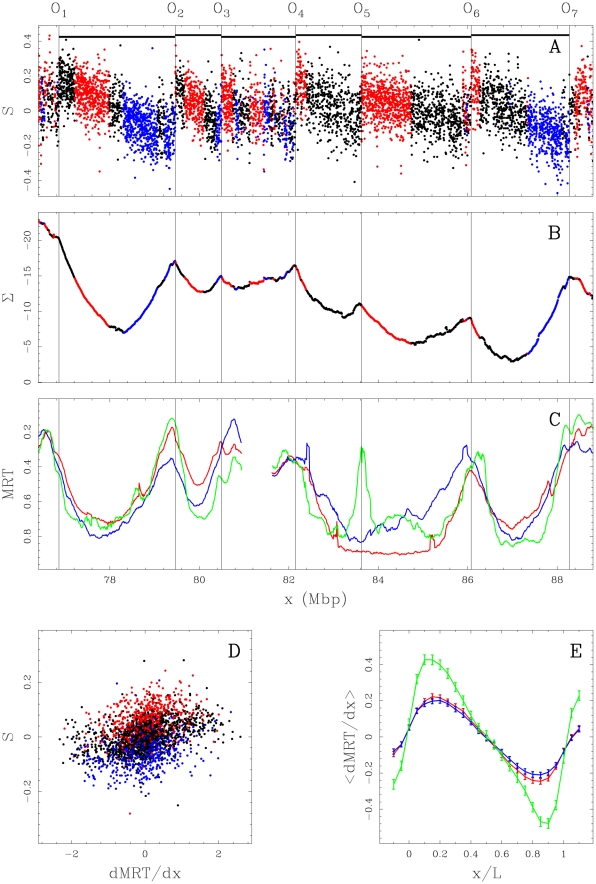
Comparing skew 
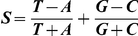
 and mean replication timing (MRT). (A) 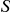 profile along a 11.4 Mb long fragment of human chromosome 10 that contains 6 skew N-domains (horizontal black bars) bordered by 7 putative replication origins 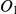 to 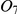. Each dot corresponds to the skew calculated for a window of 1 kb of repeat-masked sequence. The colors correspond to intergenic (black), 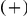 genes (red) and 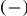 genes (blue). (B) Corresponding cumulative skew profile 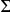 obtained by cumulative addition of 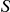-values along the sequence. (C) MRT profiles from early, 0 to late, 1 for BG02 (green), K562 (red) and GM06990 (blue) cell lines. (D) Correlations between 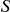 and 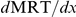, in BG02 (100 kb windows) along the 22 human autosomes; colors as in (A); the corresponding Pearson correlations are given in Table 1. (E) Average 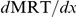 profiles (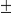 SEM) in the 663 skew N-domains after rescaling their length L to unity; colors as in (C).

Comprehensive knowledge of genetic inheritance at different development stages relies on elucidating the mechanisms that regulate the DNA spatio-temporal replication program and its possible conservation during evolution [1]. In multi-cellular organisms, there is no clear consensus sequence where initiation may occur [2], [3]. Instead epigenetic mechanisms may take part in the spatial and temporal control of replication initiation in higher eukaryotes in relation with gene expression [4]–[9]. For many years, understanding the determinants that specify replication origins has been hampered by the small number (approximately 30) of well-established replication origins in the human genome and more generally in mammalian genomes [1], [7], [10]. Recently, nascent DNA strands synthesized at origins were purified by various methods [11]–[14] to map a few hundreds putative origins in 1% of the human genome. For unclear reasons, the concordance between the different studies is very low (from 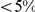 to 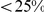) [12]–[15]. In a completely different approach to map replication origins, previous *in silico* analyses of the nucleotide compositional skew 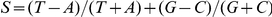 of the human genome showed that the sign of 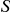 abruptly changed from 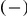 to 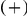 when crossing known replication initiation sites. This allowed us to predict putative origins at more than a thousand sites of 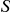 sign inversion (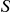-jumps) along the human genome [16], [17]. Further analyses of 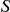 patterns identified 663 megabase-sized N-domains whose skew profile displays a N-like shape (Fig. 1A), with two abrupt 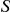-jumps bordering a DNA segment whose skew linearly decreases between the two jumps [16]–[21]. Skew N-domains have a mean length of 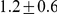 Mb and cover 29.2% of the human genome. The initiation zones predicted at N-domains borders would be specified by an open chromatin structure favorable to early replication initiation and permissive to transcription [21], [22]. The determination of HeLa replication timing profile [23] and the analysis of available timing profiles in several human cell lines [24]–[26] allowed us to confirm that significant numbers of N-domains borders harbor early initiation zones active in germline as well as in somatic cell types [18], [27].

Recent studies have shown that replication induces different mutation rates on the leading and lagging replicating strands [27]. This asymmetry of rates acting during evolution has generated the skew upward jumps that result from inversion of replication fork polarity at N-domain extremities. The skew profile along N-domains would result from superimposed effects of transcription and of replication [19], [20], [28]–[31]. Accordingly, the linear decrease of the skew (Fig. 1A) may reflect a decrease in the proportion of replicating forks propagating from the left (5′) to the right (3′) N-domain extremity. This organization of replication in a large proportion of the genome contrasts with the previously proposed segmentation of mammalian chromosomes in regions replicated either by multiple synchronous origins with equal proportion of forks coming from both directions (0.2–2.0 Mb Constant Timing Regions) or by unidirectional replication forks (0.1–0.6 Mb Transition Timing Regions) [25], [32]–[34].

Here, to determine the existence of a new type of replication domains presenting gradients of replication fork polarity, we establish (i) that the replication fork polarity and the compositional skew are proportional to each other, (ii) that the replication fork polarity can be directly extracted from the derivative of the replication timing profile. Taking advantage of replication timing profiles in several human cell types [23], , we show that the derivative of the replication timing profile of N-domains is shaped as a N. The corresponding U-shape of the replication timing profile is not specific to the germline but is generally observed in all replication timing profiles examined, thus establishing these “U-domains” as a new type of replication domains, consistent with the recent experimental observation of multiple replication initiations in most Transition Timing Regions in several human cell lines [35]. As observed with the early initiation zones bordering N-domain extremities, those specific to the U-domains are significantly enriched in open chromatin markers as well as insulator-binding proteins CTCF [36], [37] and are prone to gene activity. Analysis of recent Hi-C data [38] reveals that U-domains correspond to self-interacting structural chromatin units. These data make a compelling case that the “islands” of open chromatin observed at U-domains borders are at the heart of a compartmentalization of chromosomes into chromatin units of independent replication and of coordinated gene transcription.

## Results/Discussion

### Linking replication fork polarity to nucleotide compositional skew profile and replication timing

To establish the existence of replication domains associated with replication fork polarity gradients, we first demonstrate the relations between replication fork polarity, nucleotide compositional skew and derivative of the replication timing profile. Under appropriate hypotheses, the skew 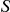 resulting from mutational asymmetries associated with replication is proportional to the fork polarity 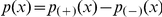 at position 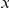 on the sequence (Material and Methods):
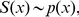
(1)where 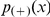 (resp. 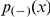) is the proportion of forks replicating in the 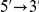 (resp. 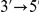) direction on the Watson strand. The linear decrease of 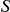 in N-domains from positive (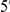 end) to negative (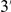 end) values thus likely reflects a linear decrease of the replication fork polarity with a change of sign in the middle of the N-domains. This result strongly supports the interpretation of N-domains (Fig. 1A–C) as the signature of a higher-order organization of replication origins in germline cells.

The replication fork polarity can also be directly deduced from replication timing data under the central hypotheses that the replication fork velocity 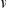 is constant and that replication is bidirectional from each origin. Note that recent DNA combing experiments in HeLa cells have shown that replication fork velocity does not significantly vary during S phase which strongly supports the former hypothesis [35]. We demonstrate that the replication fork polarity 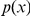 is the product of the derivative of the mean replication timing (MRT) and the replication fork velocity 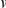 (Material and Methods):
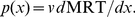
(2)The fork polarity should therefore provide a direct link between the skew 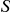 and the derivative of the replication timing profile in germline cells. To test this relationship, we used a substitute for germline MRT, the replication timing profiles of seven somatic cell lines (one embryonic stem cell, three lymphoblastoid, a fibroblast, an erythroid and HeLa cell lines) (Material and Methods). We first correlated the skew 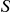 with 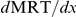, in the BG02 embryonic stem cells, over the 22 human autosomes (Fig. 1D). The significant correlations observed in intergenic (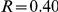, 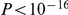), genic 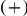 (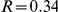, 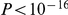) and genic 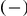 (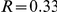, 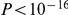) regions are representative of the correlations observed in the other 6 cell lines (Table 1). These correlations are as strong as those obtained between the 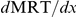 profiles in different cell lines (Supplementary Table S1), as well as those previously reported between the replication timing data themselves [26], [34], [39]. The correlations between 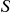 and 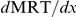 are even stronger when focusing on the 663 skew N-domains (Table 1). The correlations obtained in intergenic regions (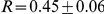) are recovered to a large extent in genic regions (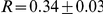) where the transcription-associated skew 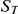 was hypothesized to superimpose to the replication-associated skew 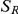 [18]–[20]. Further evidence of this link between 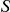 and 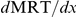 was obtained when averaging, for the different cell lines, the 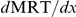 profiles inside the 663 skew N-domains after rescaling their length to unity (Fig. 1E). These mean profiles are shaped as a N, suggesting that some properties of the germline replication program associated with the pattern of replication fork polarity are shared by somatic cells.

**Table 1:** Compostional skew and derivative of the replication timing profile correlate.

R	BG02	K562	GM06990	H0287	TL010	BJ R1	BJ R2	HeLa R1	HeLa R2
GW 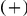	0.34	0.36	0.35	0.34	0.33	0.31	0.30	0.33	0.29
GW 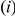	0.40	0.45	0.42	0.41	0.41	0.35	0.36	0.32	0.28
GW 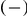	0.33	0.37	0.34	0.35	0.34	0.33	0.32	0.34	0.29
Ndom 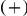	0.36	0.43	0.42	0.42	0.41	0.32	0.32	0.38	0.35
Ndom 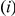	0.45	0.50	0.48	0.48	0.47	0.38	0.39	0.35	0.29
Ndom 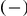	0.35	0.44	0.44	0.43	0.41	0.40	0.39	0.40	0.35

Pearson correlation (R values) between the skew 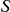 and 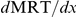, from different cell lines (Material and Methods). 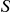 and 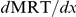 were calculated in non-overlapping 100 kb windows genome wide (GW) and in the 663 skews N-domains (Ndom). Each 100 kb window was classed as intergenic 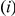, genic 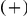 or genic 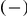 by majority rule. All p-values are 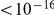.

### Replication timing U-domains are robustly observed in human cell lines

**Figure 2:**
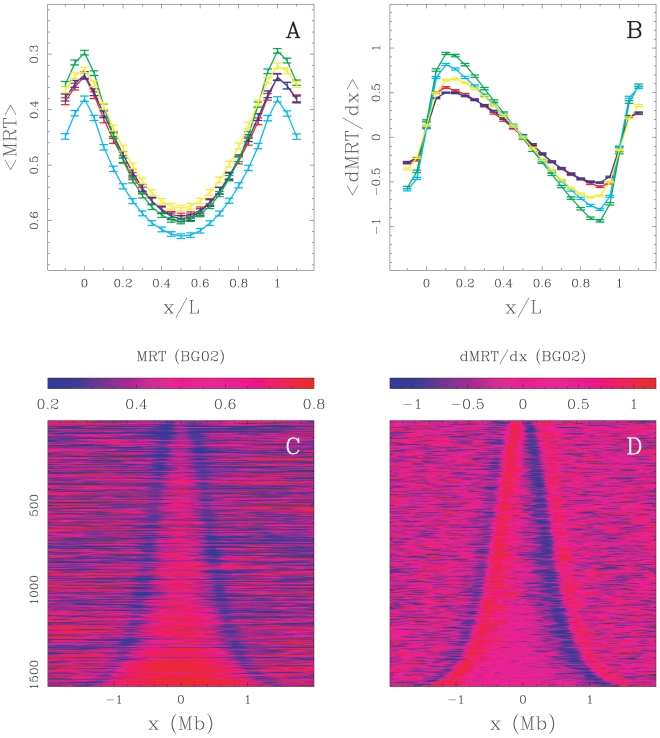
Replication timing U-domains in different human cell lines. (A) Average MRT profiles (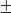 SEM) inside detected replication U-domains (Table 2). (B) Corresponding average 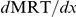 profiles (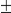 SEM). In (A) and (B), each cell line is identified by a color: BG02 (green), K562 (red), GM06990 (blue), BJ R2 (magenta), and HeLa R2 (cyan). (C) The 2534 BG02 U-domains were centered and ordered vertically from the smallest (top) to the longest (bottom). The MRT profile of each domain is figured along a horizontal line using the MRT (BG02) color map. (D) Same as in (C) but for 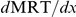 using the 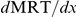 (BG02) color map.

According to Equations (1) and (2), the integration of the skew 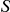 is expected to generate a profile rather similar to the replication timing profile. In segments of linearly changing skew, the integrated 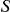 function is thus expected to show a parabolic profile. The integrated 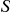 function when estimated by the cumulative skew 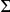 (Fig. 1B) along N-domains of a 11.4 Mb long fragment of human chromosome 10, indeed displays a U-shaped (parabolic) profile likely corresponding the replication timing profile in the germline. Remarkably, the 6 N-domains effectively correspond to successive genome regions where the MRT in the BG02 embryonic stem cells is U-shaped (Fig. 1C). The 7 putative initiation zones (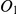 to 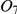) corresponding to upward 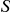-jumps (Fig. 1A), co-locate (up to the 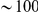 kb resolution) with MRT local extrema which supports that they are highly active in BG02. These initiation zones can present cell specificity as exemplified by the putative replication origin 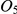 which is inactive (or late) in both the K562 erythroid and GM06990 lymphoblastoid cell lines (Fig. 1C) resulting in domain “consolidation” [40]. Two neighboring U-domains (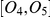 and 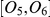) in BG02 merged into a larger U-domain in the K562 and GM06990 cell lines. Note that the other 3 N-domains (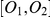,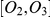, and 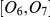) are replication timing U-domains common to BG02, K562 and GM06990. To detect U-domains in replication timing profiles at genome scale, we developed a wavelet-based method (Material and Methods, and Supplementary Text S1) which allowed us to identify in the 7 human cell lines from 664 (TL010) up to 1534 (BG02) U-domains of mean size ranging from 0.966 Mb (HeLa R2) up to 1.62 Mb (TL010) and covering from 39.6% (TL010) to 61.9% (BG02) of the genome (Table 2). For each cell line, the average MRT profile of U-domains has an expected parabolic shape (Fig. 2A) representative of individual U-domains (Fig. 2C and Supplementary Figs. S1A–S9A). Inside the U-domains, the derivative 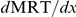 is N-shaped (Fig. 2D and Supplementary Figs. S1B–S9B) like the skew profile inside N-domains (Supplementary Figs. S1F–S9F). When rescaling the size of each U-domains to unity for a given cell line, these profiles superimpose onto a common N-shaped curve well approximated by the average 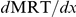 profile (Fig. 2B).

**Table 2:** Replication domains characteristics.

	Ndom	BG02	K562	GM06990	H0287	TL010	BJ R1	BJ R2	HeLa R1	HeLa R2
N	663	1534	876	882	830	664	1150	1247	1422	1498
L	1.19	1.09	1.42	1.52	1.57	1.62	1.19	1.15	1.06	0.966
G	29.2	61.9	46.1	49.5	48.1	39.6	50.5	53.2	55.7	53.5
GC	40.30	40.25	40.84	40.85	40.94	41.13	40.84	40.60	40.72	40.99

Columns corresponds to the replication timing U-domains detected in different cell lines using our wavelet-based methodology (Material and Methods, and Supplementary data) and the corresponding skew N-domains (replication domains in the germline) given for comparison. N = number, L = mean length (Mb), G = genome coverage (%), GC = mean GC-content (%) of the replication domains found in the 22 human autosomes.

To determine the amounts of U-domains conserved in different cell types, we computed for each cell type pair the mutual covering of the corresponding sets of U-domains (two U-domains are shared by two different cell lines if each domain covers more than 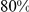 of the other domain (Table 3)). Taking as reference the matching obtained for the two BJ (68.6% and 74.3%) and HeLa (51.8% and 54.6%) cell replicates, the matchings between the other cell lines were statistically significant and comparable (from 40% to 65% for the mutual covering of lymphoblastoid cell lines). The number of U-domain shared by cell type pairs were all significantly larger than the number expected by chance (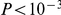, Supplementary Table S2). For example BG02 shares 197 and 189 U-domains with K562 and GM06990 respectively, when only 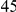 and 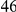 are expected by chance (Supplementary Table S3). This corresponds to a significant proportion (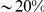) of the U-domains of the individual cell lines (Table 3), as compared to the matchings (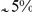) expected by chance (Supplementary Table S4). A significant percentage of N-domains correspond to U-domains (*e.g.* from 12.5% in BJ R1 up to 23.7% in BG02). This explains that when representing the MRT profile of BG02 instead of the skew 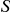, along the set of N-domains ordered according to their size, we can recognize the edges of many N-domains (Supplementary Figs. S1D–S9D). The same observation can be made when comparing the 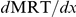 profiles (Supplementary Figs. S1E–S9E) to the corresponding skew profiles (Supplementary Fig. S1F). Note that the N-domains match only 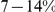 of the U-domains of various cell lines due to the very stringent N-domain selection criteria [19], [20] that yielded only 663 N-domains (29.2% of the genome) as compared to much larger U-domain numbers (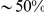 of the genome; Table 2). Replication timing U-domains are robustly observed in all cell lines, covering 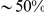 of the human genome. For each cell type, about half U-domains are shared by at least another cell line, namely BG02 (38.4%), K562 (61%), GM06990 (59.2%), BJ R1 (51.6%), HeLa R1 (44.7%). This is also true for the skew N-domains (50.2%) that likely correspond to replication timing U-domains in the germline. However about half of the genome that is covered by U-domains corresponds to regions of high replication timing plasticity where replication domains may (i) reorganize according to the so-called “consolidation” scenario (merging of two U-domains into a larger one) (Fig. 1C), (ii) experience some boundary shift and (iii) emerge in a late replicating region as previously observed in the mouse genome during differentiation [40].

**Table 3:** Correspondence between replication domains.

	Ndom	BG02	K562	GM06990	H0287	TL010	BJ R1	BJ R2	HeLa R1	HeLa R2
Ndom	100	10.2	13.6	13.5	13.1	13	7.22	8.02	8.44	8.28
BG02	23.7	100	22.5	21.4	20.5	17.9	18	19.7	16.7	15.2
K562	17.9	12.8	100	28.5	28.8	30.9	16	15.3	13.9	12.6
GM06990	17.9	12.3	28.7	100	64.6	56.2	16	15.7	12.2	12.1
H0287	16.4	11.1	27.3	60.8	100	56.6	16.9	15.5	13	11
TL010	13	7.76	23.4	42.3	45.3	100	12.3	11.9	9.21	9.21
BJ R1	12.5	13.5	21	20.9	23.4	21.2	100	68.6	23.5	20.4
BJ R2	15.1	16	21.8	22.2	23.3	22.3	74.3	100	25	22.2
HeLa R1	18.1	15.5	22.5	19.6	22.3	19.7	29	28.5	100	51.8
HeLa R2	18.7	14.9	21.5	20.5	19.9	20.8	26.6	26.6	54.6	100

Percentage of matchings between replication timing U-domains in different cell lines including skew N-domains in the germline. A U-domain in a given cell line (column) was considered as matching a U-domain in another cell line (row) if more than 80% nucleotides of each of these U-domains were common to the two domains.

### Replication timing U-domains borders are enriched in open chromatin markers

Genome-wide investigation of chromatin architecture has revealed that, at large scales (from 100 kb to 1 Mb), regions enriched in open chromatin fibers correlate with regions of high gene density [41]. Moreover there is a growing body of evidence that transcription factors are regulators of origin activation (reviewed in Kohzaki and Murakami 2005). We ask whether the remarkable genome organization observed around N-domain borders [19] is maintained around replication timing U-domain borders and to what extent it is mediated by a particular chromatin structure favorable to early replication origin specification [22].

**Figure 3:**
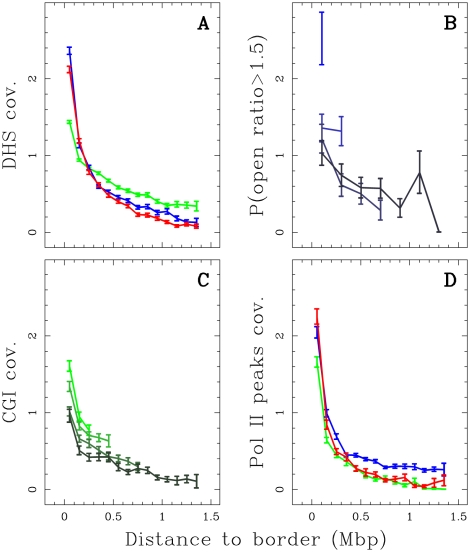
Analysis of chromatin marks along U-domains. Over representation of open chromatin markers (Material and Methods) at replication timing U-domain borders relative to the corresponding genome-wide value. (A) Mean coverage by DNase I hypersensitive zones, as a function of the distance to the closest U-domain border in BG02 using DNase H1-hESC data (green, genome-wide mean value = 0.0073), K562 using DNase K562 data (red, genome-wide mean value = 0.0138), GM06990 using DNase GM06990 data (blue, genome-wide mean value = 0.0107). (B) Proportion of clones presenting a ratio of “open” over input chromatin greater than 1.5 versus the distance to the closest U-domain border in GM06990 for four U-domain size categories: L
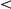
0.8 Mb, 0.8 Mb
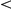
L
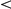
1.2 Mb, 1.2 Mb
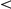
L
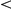
1.8 Mb and 1.8 Mb
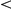
L
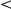
3 Mb from light to dark blue curves (genome-wide mean value = 0.20). (C) Mean coverage by 1 kb-enlarged CpG islands as a function of the distance to the closest U-domain border in BG02 for the four U-domain size categories defined in (B) from light to dark green curves (genome-wide mean value = 0.0254). (D) Mean coverage by Pol II peaks as a function of the distance to the closest U-domain border in BG02 (green: Pol II in H1 ESC, genome-wide mean value = 0.0026), K562 (red: Pol II in K562, genome-wide mean value = 0.0024), GM06990 (blue: Pol II in GM12878, genome-wide mean value = 0.0097).

When mapping DNase I sensitivity data (Material and Methods) [42] on the U-domains, we observed that the mean coverage is maximal at U-domain extremities and decreases significantly from the extremities to the center that is rather insensitive to DNase I cleavage (Fig. 3A and Supplementary Fig. S10). This decrease, from values significantly higher than the genome-wide average value, extends over 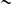
150 kb, whatever the size of the replication timing U-domain (Supplementary Fig. S11A–C) suggesting that, for all examined cell lines, early replicating U-domains borders are at the center of 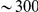 kb wide open chromatin regions. We observed a significant anti-correlation between DNase I cleavage sensitivity data and replication timing data in BG02 (DNase H1-hESC: 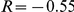, 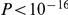), K562 (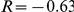, 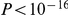) and GM06990 (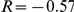, 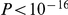) cell lines as well as in the other four cell lines (data not shown; note that this was still observed when controlling for the GC content). This is further supported by open over input chromatin ratio data obtained from human lymphoblastoid cells [41]. We observed that the regions presenting an open/input ratio 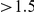 also decreased significantly (3-fold) from U-domain borders to centers (Fig. 3B).

Cytosine DNA methylation is a mediator of gene silencing in repressed heterochromatic regions, while in potentially active open chromatin regions, DNA is essentially unmethylated [43]. DNA methylation is continuously distributed over mammalian chromosomes with the notable exception of CpG islands (CGIs) and in turn of certain CpG rich promoters and transcription start sites (TSSs). Along the observation that the hypomethylation level of CGIs extends to about 1 kb in flanking regions, we used 1 kb-enlarged CGI coverage as an hypomethylation marker (Material and Methods) [22]. When averaging over the U-domains detected in BG02, we robustly observed a maximum of CGI coverage at U-domain borders as the signature of hypomethylation and a decrease over a characteristic distance of 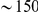 kb (Fig. 3C), similar to what we found for DNase I sensitivity coverage (Fig. 3A). This contrasts with the GC-content profile that strongly depends on the U-domain size and decreases very slowly toward the U-domain center without exhibiting any characteristic scale (Supplementary Fig. S11D–F). These observations are consistent with the hypothesis that early replication origins at U-domain borders are associated with CGIs that are possibly protected from methylation by colocalization with replication origins [44].

Open chromatin markers have been associated with genes. For example 16% of all DNase I hypersensitive sites (HS) are in the first exon or at the TSS of a gene and 42% are found inside a gene [45]. Also, more than 90% of broadly expressed housekeeping genes have a CpG-rich promoter [46]. Remarkably, the mean profiles of Pol II binding Chip-Seq tag density (Material and Methods) along U-domains detected in BG02, K562 and GM06990 cell lines strongly decay over 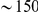 kb away from U-domain borders (Fig. 3D). This indicates that, whatever the cell line, the open chromatin regions around replication U-domains are prone to transcription whereas U-domain central regions appear, on average, transcriptionally silent.

Importantly, we have reproduced the analyses of open chromatin markers near U-domain borders that do not match with a N-domain border (at 100 kb resolution) and confirmed that the results reported in Fig. 3 apply to the initiation zones at U-domains borders of every cell line (Supplementary Fig. S12).

### Replication timing U-domains are insulated compartments of genome-wide chromatin interactions (Hi-C)

**Figure 4:**
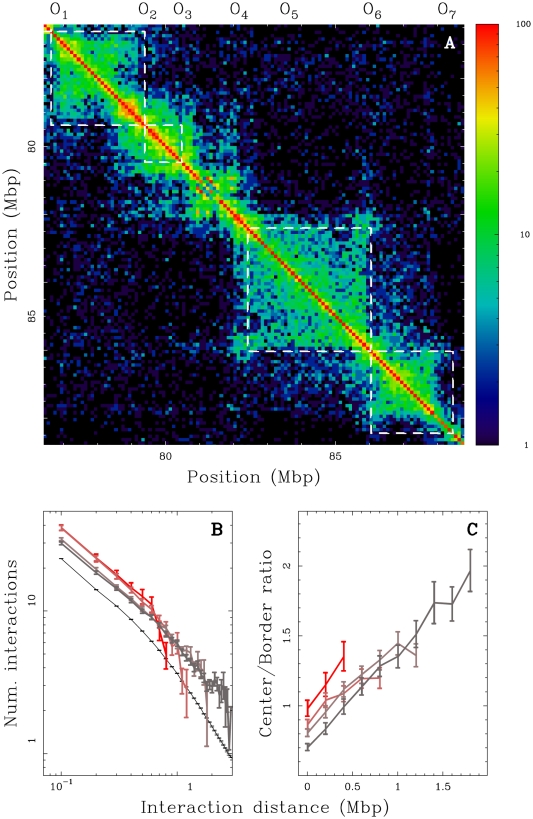
Chromatin conformation data and U-domain compartmentalization of the genome. (A) Hi-C proximity matrix corresponding to intrachromosome interactions on the 11.4 Mb long fragment of human chromosome 10 (Fig. 1), as measured in the K562 cell line (Material and Methods). Each pixel represents all interactions between a 100 kb locus and another 100 kb locus; intensity corresponding to the total number of reads is color coded according to the colormap (right). The dashed squares correspond to replication timing U-domains detected in the K562 cell line. (B) Number of interactions between two 100 kb loci versus the distance separating them (logarithmic scales) as computed genome wide (black) or in K562 replication U-domains only, for four U-domain size categories: L
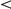
0.8 Mb, 0.8 Mb
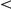
L
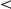
1.2 Mb, 1.2 Mb
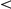
L
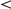
1.8 Mb and 1.8 Mb
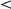
L
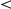
3 Mb (from light to dark red). (C) Ratio of the number of interactions between two 100 kb loci inside the same U-domain at equal distance from its center and the number of interactions between loci on opposite sides and equal distance from a U-domain border, versus the distance between them; colors as in (B).

**Figure 5:**
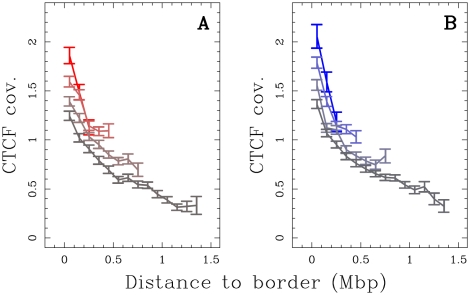
Enrichment in insulator-binding protein CTCF at replication U-domains borders. (A) Mean coverage by CTCF enriched signals versus the distance to the closest U-domain border in K562 cell line for four U-domain size categories: L
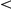
0.8 Mb, 0.8 Mb
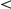
L
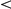
1.2 Mb, 1.2 Mb
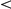
L
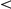
1.8 Mb and 1.8 Mb
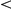
L
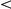
3 Mb, from light to dark red curves (genome-wide mean value = 0.0051). (B) Same as in (A) but for the GM06990 cell line (blue code shades) (genome-wide mean value = 0.0046).

It is widely recognized that the 3D chromatin tertiary structure provides some understanding to the experimental observation of the so-called replicon and replication foci [2], [47]. In particular, replicon size, which is dictated by the spacing between active origins, correlates with the length of chromatin loops [8], [47], [48]. The chromosome conformation capture technique [38] has provided access to long-range chromatin interactions as a footprint of the different levels of chromatin folding in relation with gene activity and the functional state of the cell. From a comparative analysis of replication timing data and Hi-C data correlation matrix in the human genome, some dichotomic picture has been proposed where early and late replicating loci occur in separated compartments of open and closed chromatin respectively [34], [38]. Here, instead of considering the partitioning of the chromosomes derived from all intrachromosomal interactions of each locus (using a principal component of the principal component analysis of the Hi-C data over each chromosome), we focused on interactions between loci separated by short genomic distances (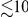 Mb) over which the contact probabilities are the highest [38]. First, we performed this zoom in the Hi-C contact matrix in the K562 cell line at the 100 kb resolution (Material and Methods) for the 11.4 Mb fragment of human chromosome 10 which contains four U-domains in K562 (Fig. 1; 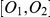, 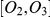, 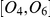 and 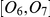). We found that these four U-domains remarkably correspond to four matrix square-blocks of enriched interactions (Fig. 4A). Hence, we recover that early replicating zones that border a U-domain (*e.g.* 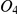 and 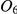 separated by 3.9 Mb), have a high contact probability as the signature of 3D spatial proximity. However, we also observe a high contact probability of the two early replicating borders with the late replicating U-domain center and interactions appear sparse for loci in separate U-domains (*e.g.* 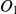 and 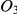 separated by 3.6 Mb). Further examination of the average behavior of intrachromosomal contact probability as a function of genomic distance for the complete genome corroborates these observations. We found that the mean number of interactions between two 100 kb loci of the same U-domain decays when increasing their distance as observed genome-wide (Fig. 4B). Importantly, the mean number of pairwise interactions is significantly higher inside the U-domains than genome-wide and this seems to depend on the U-domain length. In particular, we found that the smaller the domain, the higher the mean number of interactions which is probably a signature of a more open chromatin structure. When comparing the contact probability between two loci inside a U-domain or lying in neighboring U-domains (Fig. 4C), we observed that the latter is higher than the former for distances smaller than the characteristic size (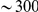 kb) of the open chromatin structure at U-domain borders (Fig. 3). Above this characteristic distance, the tendency is reversed and the ratio increases up to 2 for distances 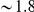 Mb (Fig. 4C). These data suggest that the segmentation of the genome into replication timing U-domains corresponds to some spatial compartmentalization into self-interacting structural chromatin units insulated by two boundaries of open, accessible, actively transcribed chromatin. This conclusion is strengthened by the observation that U-domain borders are significantly enriched in the insulator binding protein CTCF (Fig. 5), that is known to be involved in chromatin loop formation conditioning communication between transcriptional regulatory elements [36], [37], [49], [50]. Quantitatively similar results were obtained for the lymphoblastoid GM06990 cell line for which both replication timing and Hi-C data were available (Supplementary Fig. S13).

### Perspectives

The mapping of open chromatin marks along U-domains revealed that they are bordered by early replication initiation zones likely specified by a 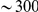 kb wide region of accessible, open chromatin permissive to transcription. Such a strong gradient of open chromatin environment was not observed around a large fraction of the 283 replication origins identified in ENCODE regions [12]; only 29% overlap a DNase I hypersensitivity site and half of them do not present open chromatin marks and are not associated with active transcription [22]. Furthermore, the typical inter-origin distance in human cells is 50–100 kb [12], [4], a much smaller value than the mean U-domain size (1–1.5 Mb). These data can be reconciled in a model [51], [52] where replication origins fire independently and their properties (intrinsic firing time probability, efficiency) are specified by the chromatin state: efficient early replicating origins in euchromatic regions (U-domains borders) and late replicating or less efficient origins in heterochromatic regions (U-domains centers). A more dynamical model can also be proposed in which replication first initiates at U-domain borders followed by a chromatin gradient-mediated succession of secondary origin activations. These origins may be remotely activated by the approach of a center-oriented fork that may stimulate initiation due to changes in DNA supercoiling in front of the fork or to association of chromatin remodelers or origin triggering factors with replication fork proteins [35]. This “domino” model could explain why replication progresses from U-domain borders much faster (3–5 times) than the known speed of single fork [8], [35], [48]. Indeed the U-shape of the replication timing profile indicates that the replication wave accelerates (effective velocity equals the inverse of the replication timing derivative, Equation (2)) as the signature of an increasing origin firing frequency during the S-phase [53]. It will be essential to determine to what extent the chromatin state influences fork progression and origins activations and whether outside of U-domains, the genome replicates according to a similar or completely different scenario.

## Materials and Methods

### Linking nucleotide compositional skew to replication fork polarity

We use the formalism of Markov processes to prove that replication-associated asymmetries between the substitution rates of the two DNA strands induce, in the limit of small asymmetries, a nucleotide compositional skew proportional to the replication fork polarity (the average direction of a locus' replication). Models of DNA composition evolution are usually written in the form of an autonomous and homogeneous system of first-order differential equations [54]:
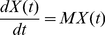
(3)where 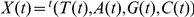 is the vector which represents the state of the system, *i.e.* for 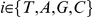, 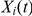 is the frequency of 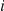 at time 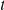, and for 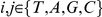, 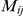 is the substitution rate of 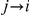. A general and well-known property of a Markov process like Equation (3) is that 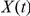 tends exponentially towards the equilibrium value 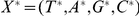, defined as 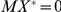. The evolution on the complementary strand is given by the same equation but for 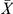 and 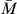, 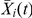 defines the frequency vector on the complementary strand, 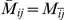 is the substitution rate matrix on the complementary strand, and 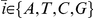 denotes the complementary base of 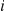. Under no-strand-bias conditions [55], the same substitution rates affect the two strands, *i.e.* 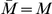 leading to the so-called parity rule of type 2 (PR2): 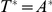 and 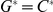 [56]–[59]. Departure from this symmetry condition can thus be quantified by decomposing 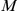 into symmetric 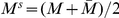 and antisymmetric 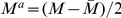 parts, the latter accounting for the establishment of a nucleotide compositional strand asymmetry during evolution.

According to our previous studies of the skew 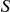 in mammalian genomes [16]–[20],[29]–[31], we can reasonably suppose that replication and transcription are the main mechanisms responsible for deviations in PR2. If we concentrate on the effect of replication on DNA composition, we may consider intergenic regions only: then the substitution rate matrix 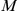 can be written as
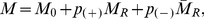
(4)where 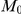 is a substitution rate matrix satisfying the no-strand bias conditions (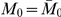), 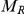 is the substitution rate matrix associated with replication and 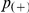 (resp. 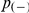) the proportion of forks replicating the region of interest in the 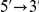 (resp. 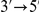) direction. 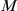 can be easily decomposed into a symmetric part:
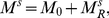
(5)and an antisymmetric part:
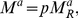
(6)which turns out to be proportional to the fork polarity:
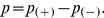
(7)


Under the assumption that 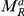 is significantly smaller than 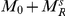, namely
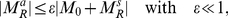
(8)we can use perturbation theory to solve Equation (3) and to show that if the compositional skews:
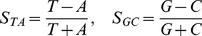
(9)are initially null (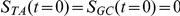), then the total skew will be proportional to the fork polarity 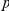 at all times 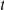 up to terms of order 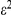 (Equation (8)):
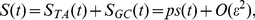
(10)where 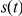 is a function that depends only on 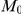 and 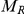. Using the mean nucleotide substitution rate matrix 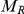 computed in the intergenic regions on each side (300 kb windows) of the 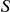-upward jumps [27], the coefficients of 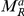 were found to be much smaller than those of 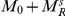 with 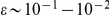 (Supplementary Text S1). Thus, according to Equation (10), the observed linear decrease of the skew 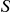 in N-domains from positive (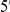 end) to negative (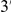 end) values likely reflects the progressive linear decrease of the replication fork polarity with a change of sign in the middle of the skew N-domains. These results provide strong support to the interpretation of skew N-domains (Fig. 1A) as independent replication units in germline cells.

### Determining the replication fork polarity from replication timing data

**Figure 6:**
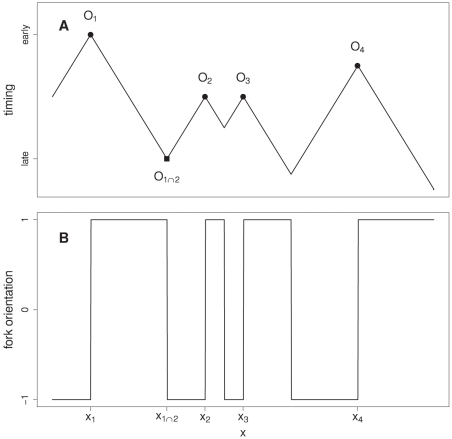
Modeling the spatio-temporal replication program. Replication timing 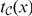 (A) and fork orientation 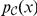 (B) of the configuration 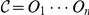 where 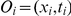 corresponds to the origin 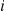 positioned at location 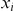 and firing at time 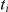. Fork coming from 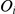 meets the fork coming from 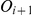 at the space-time point 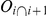 defined in Equation (11). The replication timing and fork orientation at the spatial position 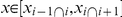 are given by Equation (12) from which we deduce the relationship 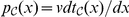 and in turn Equation (14) for the replication fork polarity and the derivative of the MRT. In this picture of the spatio-temporal replication program, the replication fork velocity 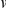 is assumed to be constant and replication is bidirectional from each origin.

As previously pointed out in [52], the derivative of the replication timing profile does not provide a direct estimator of the replication fork velocity as it also depends on the fork polarity. Here, we demonstrate that the replication fork polarity can be directly deduced from replication timing data under the central hypothesis that the replication fork speed 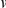 is constant and that replication is bidirectional from each origin. For a given cell cycle, let 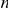 be the number of activated origins, 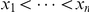 their positions along the genome and 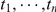 their initiation times. Then the configuration 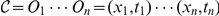 (where and when the origins of replication fire during the S-phase) completely specifies the spatio-temporal replication program (Fig. 6) [51], [52]. If we denote 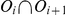 the event “the fork coming form 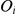 meets the fork coming from 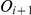
” whose space-time coordinates are:
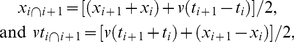
(11)then the replication timing and fork orientation 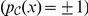 at spatial position 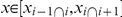 are given by (Fig. 6):
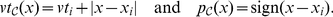
(12)We clearly see that since 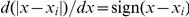 then the fork orientation is equal to 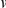 times the derivative of the replication timing:
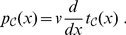
(13)Under the hypothesis of constant fork velocity 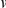, this relationship holds in whole generality in each cell cycle and at every locus 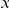 without any specific asumption on the distribution of initiation events. By definition, the replication fork polarity is the population average over cell cycles of the fork orientation: 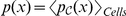. Hence, when averaging over cell cycles, Equation (13) yields:
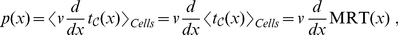
(14)where we have used the fact that the spatial derivative commutes with the population average and that by definition 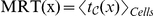. The replication fork polarity therefore provides a direct link between the skew 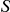 and the derivative of the MRT (Equations (10) and (14)) in germline cells.

### Sequence and annotation data

Sequence and annotation data were retrieved from the Genome Browsers of the University of California Santa Cruz (UCSC) [60]. Analyses were performed using the human genome assembly of March 2006 (NCBI36 or hg18). As human gene coordinates, we used the UCSC Known Genes table. When several genes presenting the same orientation overlapped, they were merged into one gene whose coordinates corresponded to the union of all the overlapping gene coordinates, resulting in 23818 distinct genes. We used CpG islands (CGIs) annotation provided in UCSC table “cpgIslandExt”.

### Replication N-domains

The coordinates of the 678 human replication N-domains for assembly NCBI35/hg17 were obtained from the authors [19] and mapped using LiftOver to hg18 coordinates; we kept only the 663 N-domains that had the same size after conversion.

### Determining mean replication timing profiles

We determined the mean replication timing profiles along the complete human genome using Repli-Seq data [23], [26] (Supplementary Text S1, and Supplementary Fig. S14). For embryonic stem cell line (BG02), three lymphoblastoid cell lines (GM06990, H0287, TL010), a fibroblast cell line (BJ, replicates R1 and R2), and erythroid K562 cell line, Repli-Seq tags for 6 FACS fractions were downloaded from the NCBI SRA website (Studies accession: SPR0013933) [26]. For the HeLa cell line we computed the mean replication timing (MRT) instead of computing the S50 (median replication timing) as in [23].

### Detection of U-domains along mean replication timing profiles

We developed a segmentation method of the MRT profile into U-domains based on the continuous wavelet transform. This method amounts to perform objective (U-) pattern recognition in 1D signals where the U-motif is picked out from the background signal variations (Supplementary Text S1, and Supplementary Fig. S15).

### Correlation analysis

For the analysis of correlations, we reported the Pearson's product moment correlation coefficient 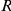 and the associated P-value for no association (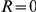). All statistical computations were performed using the R software (http://www.r-project.org/).

### DNase I hypersensitive site data

We used the DNaseI sensitivity measured genome-wide [42]. Data corresponding to Release 3 (Jan 2010) of the ENCODE UW DNaseI HS track, were downloaded from the UCSC FTP site: ftp://hgdownload.cse.ucsc.edu/goldenPath/hg18/encodeDCC/wgEncodeUwDnaseSeq/.

We plotted the coverage by DNase Hypersentive Sites (DHSs) identified as signal peaks at a false discovery rate threshold of 0.5% within hypersensitive zones delineated using the HotSpot algorithm (“wgEncodeUwDnaseSeqPeaks” tables). When several replicates were available, data were merged.

### Genome-wide maps of Pol II and CTCF binding

We used ChIP-seq data using antibody for Pol II and CTCF from Release 3 (Mar 2010) of the ENCODE Open Chromatin track [11], [61]. Data were downloaded from the UCSC FTP site: ftp://hgdownload.cse.ucsc.edu/goldenPath/hg18/encodeDCC/wgEncodeChromatinMap.

We plotted coverage by regions of enriched signal in ChIP experiments, called based on signals created using F-Seq [62] (“wgEncodeUtaChIPseqPeaks” tables). Significant regions were determined at an approximately 95% sensitivity level. We always used the most recent version of data.

### Whole genome chromatin conformation data

We used the spatial proximity maps of the human genome generated using Hi-C method [38]. We downloaded 100 kb resolution maps for GM06990 and K562 cell lines from the GEO web site (GSE18199_binned_heatmaps): http://www.ncbi.nlm.nih.gov/geo/query/acc.cgi?acc=GSE18199.

### Chromatin fiber density data

Open over input chromatin ratio data from human lymphobastoid cells were obtained from the authors [41].

### Data availability

Coordinates of N-domains and U-domains in the investigated 7 cell lines can be downloaded from: http://perso.ens-lyon.fr/benjamin.audit/ReplicationDomainsPLoSComputBiol2012/.

## Supporting Information

Figure S1The 1534 replication timing U-domains detected in BG02 embryonic stem cells were centered and ordered vertically from the smallest (top) to the largest (bottom) : the MRT (A), dMRT/dx (B), and skew 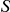 (C) profiles of each domain are figured along a horizontal line using the corresponding color maps. Same representation of the MRT (D), dMRT/dx (E), and 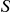 (F) profiles in the 663 skew N-domains.(PDF)

Figure S2 Same as in Supplementary Fig. S1 but for the erythroid K562 cell line (876 replication timing U-domains).
(PDF)

Figure S3 Same as in Supplementary Fig. S1 but for the lymphoblastoid GM06990 cell line (882 replication timing U-domains).
(PDF)

Figure S4 Same as in Supplementary Fig. S1 but for the lymphoblastoid H0287 cell line (830 replication timing U-domains).
(PDF)

Figure S5 Same as in Supplementary Fig. S1 but for the lymphoblastoid TL010 cell line (664 replication timing U-domains).
(PDF)

Figure S6 Same as in Supplementary Fig. S1 but for the fibroblast BJ cell line (Replicate experiment 1 ∶ 1150 replication timing U-domains).
(PDF)

Figure S7 Same as in Supplementary Fig. S1 but for the fibroblast BJ cell line (Replicate experiment 2 ∶ 1247 replication timing U-domains).
(PDF)

Figure S8 Same as in Supplementary Fig. S1 but for the HeLa cell line (Replicate experiment 1 ∶ 1422 replication timing U-domains).
(PDF)

Figure S9 Same as in Supplementary Fig. S1 but for the HeLa cell line (Replicate experiment 2 ∶ 1498 replication timing U-domains).
(PDF)

Figure S10 Mean coverage (relative to the genome average) by DNase I hypersensitive zones, as a function of the distance to the closest U-domain border in H0287 (blue solid line : DNase GM06990, genome-wide mean value = 0.0107), in TL010 (blue dashed line : DNase GM06990, genome-wide mean value = 0.0107), in BJ R1 (light blue solid line : DNase BJtert, genome-wide mean value = 0.0164), in BJ R2 (light blue dashed line : DNase BJtert, genome-wide mean value = 0.0164), in HeLa R1 (magenta solid line : DNase HeLa S3, genome-wide mean value = 0.0136), in HeLa R2 (magenta dashed line : DNase HeLa S3, genome-wide mean value = 0.0136).
(PDF)

Figure S11Mean coverage (relative to the genome average) of DNase I hypersensitive zones (A–C) and GC content (D–F) as a function of the distance to the closest U-domain border in K562 (A,D), GM06990 (B,E) and BG02 (C,F), for four U-domain size categories : L
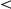
0.8 Mb, 0.8 Mb
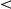
L
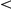
1.2 Mb, 1.2 Mb
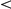
L
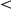
1.8 Mb and 1.8 Mb
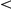
L
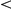
3 Mb from light to dark curves.(PDF)

Figure S12 Same analysis as in Fig. 3 but restricted to replication timing U-domain borders that do not colocate within 100 kb with a N-domain border.
(PDF)

Figure S13(A) 
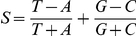
 profile along a 23 Mb long fragment of human chromosome 5 that contains 5 detected skew N-domains (black horizontal bars). Each dot corresponds to the skew calculated for a window of 1 kb of repeat-masked sequence. The colors correspond to intergenic (black), 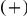 genes (red) and 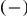 genes (blue). (B) MRT profile from GM06990 cell line (blue curve); the vertical dashed blue lines correspond to the edges of 10 detected replication timing U-domains (horizontal blue bars). (C) Hi-C proximity matrix corresponding to intrachromosome interactions on the corresponding 23 Mb long fragment of human chromosome 5, as measured in the GM06990 cell line (Methods). Each pixel represents all interactions between a 100 kb locus and another 100 kb locus; intensity corresponding to the total number of reads is color coded according to the colormap (right). The dashed squares correspond to the 10 detected U-domains. (D) Number of interactions between two 100 kb loci versus the distance separating them (logarithmic scales) as computed genome wide (black) or in replication U-domains only, for four U-domain size categories : L
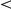
0.8 Mb, 0.8 Mb
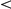
L
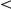
1.2 Mb, 1.2 Mb
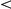
L
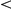
1.8 Mb and 1.8 Mb
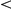
L
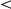
3 Mb (from light to dark blue). (E) Ratio of the number of interactions between two 100 kb loci that are inside the same U-domain at equal distance from its center and the number of interactions between loci in different U-domains at equal distance from a U-domain border, versus the distance between them (logarithmic scales); the color coding is the same as in (D). The number of interactions per pair of 100 kb loci corresponds to averaging over the 882 U-domains detected in the GM06990 cell line (Table 2).(PDF)

Figure S14(A) Normalized tag densities on a 25 Mb long fragment of chromosome 10, for the GM06990 cell line, and the corresponding computed MRT (white line). (B) “Denoised” normalized tag densities on the same genomic fragment and the corresponding MRT (white line). In (A) and (B) the tag densities for each S-phase fraction (G1–G2) are color coded using the color map situed at the top. (C) Comparison on the same genomic fragment of the MRT computed on the normalized tag densities (cyan line) and the MRT computed on the “denoised” normalized tag densities (blue line). (D) Probability density function (P.d.f.) of the genome-wide distribution of the normalized tag densities for each S-phase fraction from G1 to G2 from bottom to top (black histogram). The mode 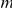 of the distribution is given by the red bar, the threshold 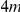 used for denoising is given by the green bar.(PDF)

Figure S15(A) MRT profile obtained in K562 cell line along a 11.4 Mbp long segment of human chromosome 10. (B) Space-scale representation of second-order variations for the MRT profile presented in (A); 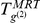 (Equation (S7)) values are color coded using green (resp. orange) shades for negative (resp. positive) curvature (note that MRT axis is going downwards). Horizontal dashed line marks scale 300 kb used to detect regions of preferential replication initiation (vertical lines). Pairs of horizontal bars delineate the scale range where strong negative curvature is expected for parabolic U-shaped MRT profile. Regions delineated by two successive regions of preferential replication initiation are kept as U-domain if 
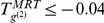
 at their midpoint for some scale value in this range.(PDF)

Table S1Pearson correlation (R values) of the derivative of MRT, dMRT/dx, between different pairs of human cell lines (Methods). dMRT/dx was calculated in non-overlapping 100 kb windows over the 22 human autosomes. All p-values are 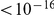.(PDF)

Table S2 Number of matchings between replication timing U-domains in different pairs of cell lines including skew N-domains in the germline. A U-domain in a given cell line (column) was considered as matching a U-domain in another cell line (row) if more than 80% nucleotides of each of these U-domains were common to the two domains.
(PDF)

Table S3 Number of matchings between randomly re-positioned replication timing U-domains in different pairs of cell lines including skew N-domains in the germline (1000 simulations were used to obtain the mean values). A U-domain in a given cell line (column) was considered as matching a U-domain in another cell line (row) if more than 80% nucleotides of each of these U-domains were common to the two domains.
(PDF)

Table S4 Percentage of matchings between randomly re-positioned replication timing U-domains in different pairs of cell lines including skew N-domains in the germline (1000 simulations were used to obtain the mean values). A U-domain in a given cell line (column) was considered as matching a U-domain in another cell line (row) if more than 80% nucleotides of each of these U-domains were common to the two domains.
(PDF)

Text S1 Supplementary methods: (i) Substitution rate matrix associated to replication (ii) Determination of mean replication timing profiles from experimental data and (iii) Detection of U-domains along mean replication timing profiles.
(PDF)
