# Supplementary material for: Replication Fork Polarity Gradients Revealed by Megabase-Sized U-Shaped Replication Timing Domains in Human Cell Lines
Source: PLoS Comput Biol. 2012 Apr 5;8(4):e1002443. doi: 10.1371/journal.pcbi.1002443 (PMC3320577; doi:10.1371/journal.pcbi.1002443)
Supplement: Figure S1 — The 1534 replication timing U-domains detected in BG02 embryonic stem cells were centered and ordered vertically from the smallest (top) to the largest (bottom) : the MRT (A), dMRT/dx (B), and skew (C) profiles of each domain are figured along a horizontal line using the corresponding color maps. Same representation of the MRT (D), dMRT/dx (E), and (F) profiles in the 663 skew N-domains. (PDF) [file pcbi.1002443.s001.pdf]

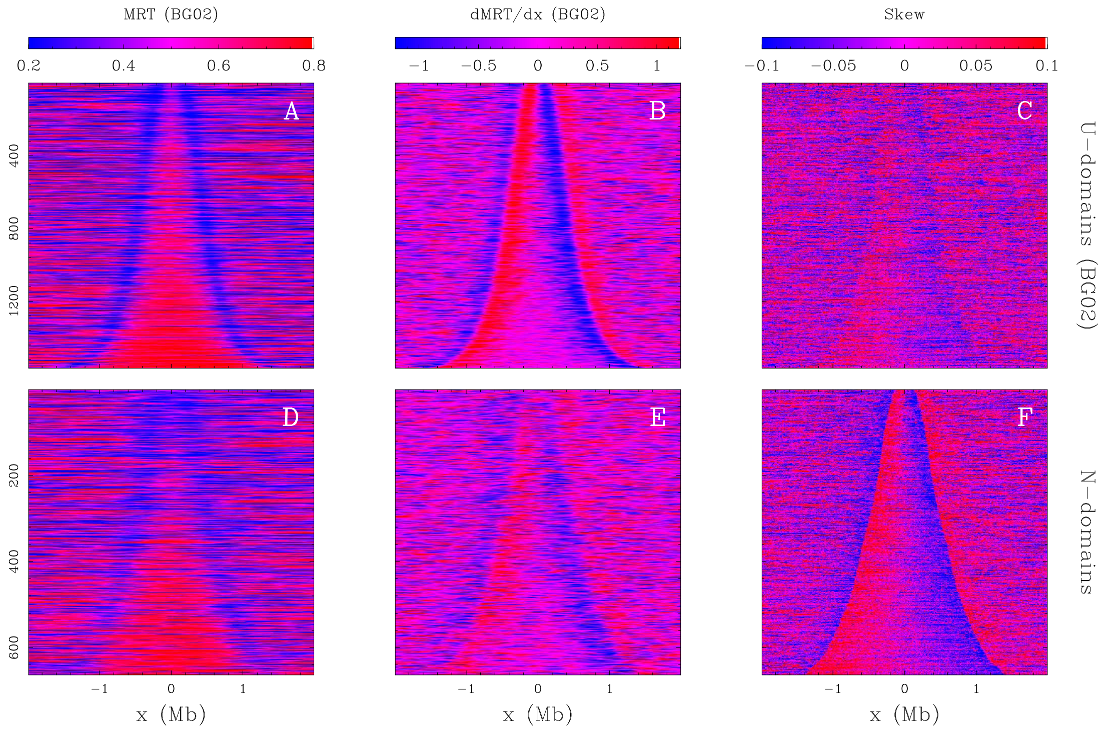

Figure S1. The 1534 replication timing U-domains detected in BG02 embryonic stem cells were centered and ordered vertically from the smallest (top) to the largest (bottom) : the MRT (A), dMRT/dx (B), and skew S (C) profiles of each domain are figured along a horizontal line using the corresponding color maps. Same representation of the MRT (D), dMRT/dx (E), and S (F) profiles in the 663 skew N-domains.
