# Supplementary material for: Replication Fork Polarity Gradients Revealed by Megabase-Sized U-Shaped Replication Timing Domains in Human Cell Lines
Source: PLoS Comput Biol. 2012 Apr 5;8(4):e1002443. doi: 10.1371/journal.pcbi.1002443 (PMC3320577; doi:10.1371/journal.pcbi.1002443)
Supplement: Figure S2 — Same as in Supplementary Fig. S1 but for the erythroid K562 cell line (876 replication timing U-domains). (PDF) [file pcbi.1002443.s002.pdf]

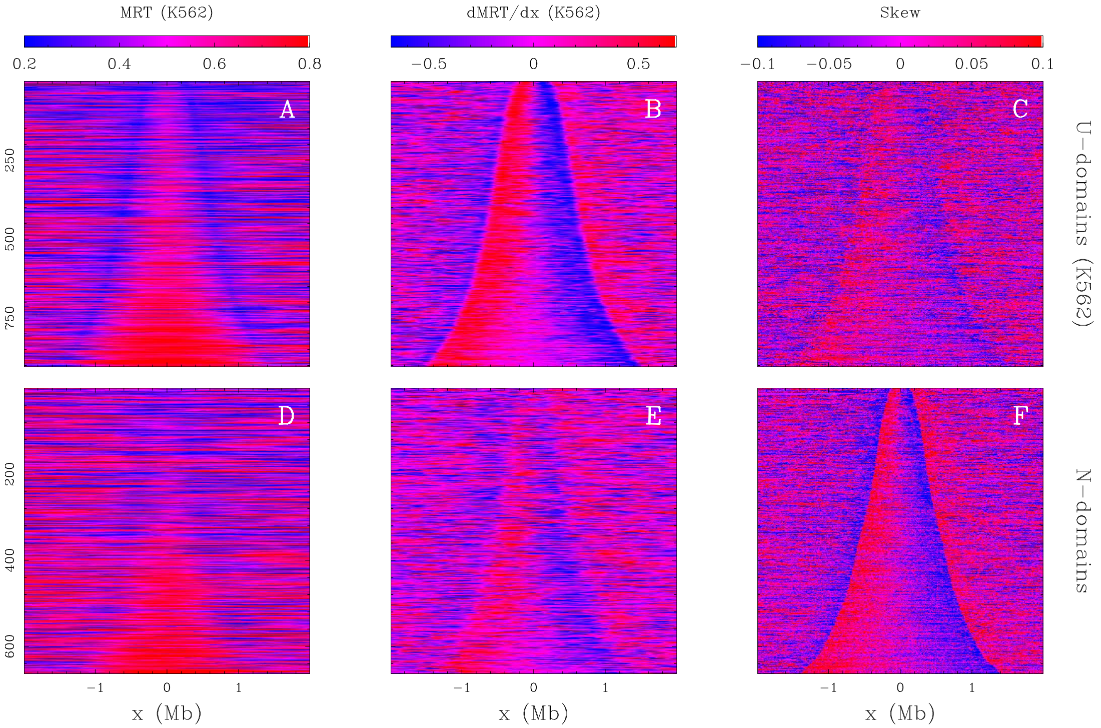

Figure S2. Same as in Supplementary Fig. S1 but for the erythroid K562 cell line (876 replication timing U-domains).
