# Supplementary material for: Replication Fork Polarity Gradients Revealed by Megabase-Sized U-Shaped Replication Timing Domains in Human Cell Lines
Source: PLoS Comput Biol. 2012 Apr 5;8(4):e1002443. doi: 10.1371/journal.pcbi.1002443 (PMC3320577; doi:10.1371/journal.pcbi.1002443)
Supplement: Figure S7 — Same as in Supplementary Fig. S1 but for the fibroblast BJ cell line (Replicate experiment 2 ∶ 1247 replication timing U-domains). (PDF) [file pcbi.1002443.s007.pdf]

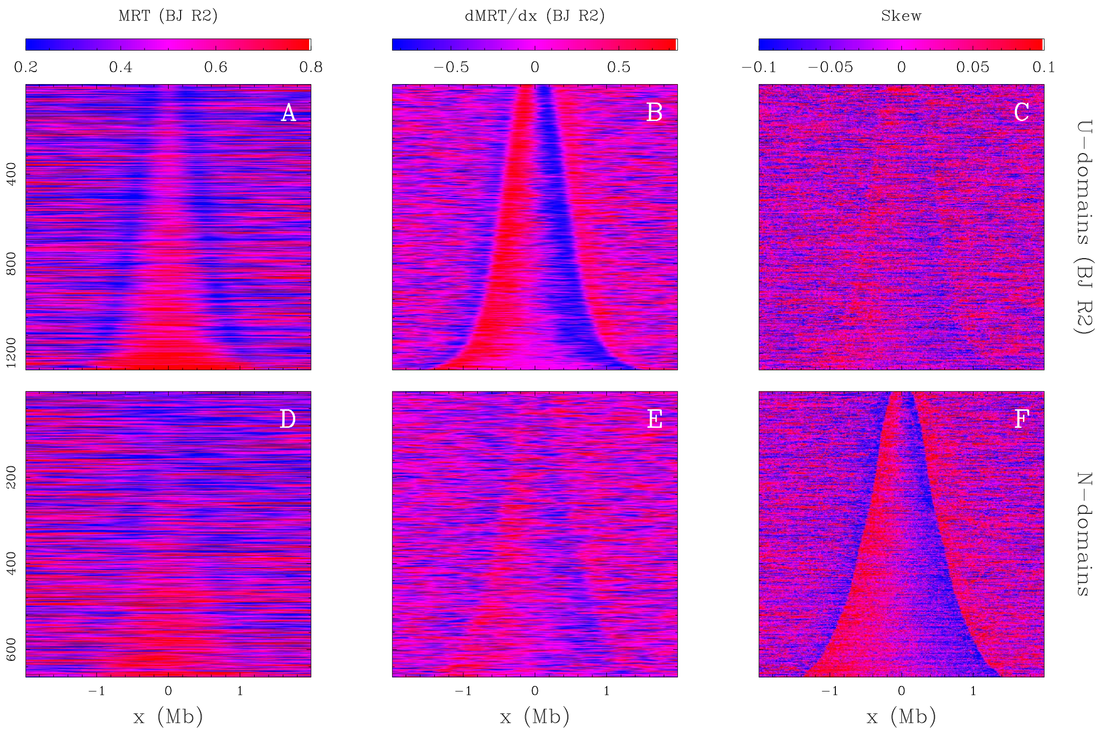

Figure S7. Same as in Supplementary Fig. S1 but for the fibroblast BJ cell line (Replicate experiment 2 : 1247 replication timing U-domains).
