# Supplementary material for: Replication Fork Polarity Gradients Revealed by Megabase-Sized U-Shaped Replication Timing Domains in Human Cell Lines
Source: PLoS Comput Biol. 2012 Apr 5;8(4):e1002443. doi: 10.1371/journal.pcbi.1002443 (PMC3320577; doi:10.1371/journal.pcbi.1002443)
Supplement: Figure S9 — Same as in Supplementary Fig. S1 but for the HeLa cell line (Replicate experiment 2 ∶ 1498 replication timing U-domains). (PDF) [file pcbi.1002443.s009.pdf]

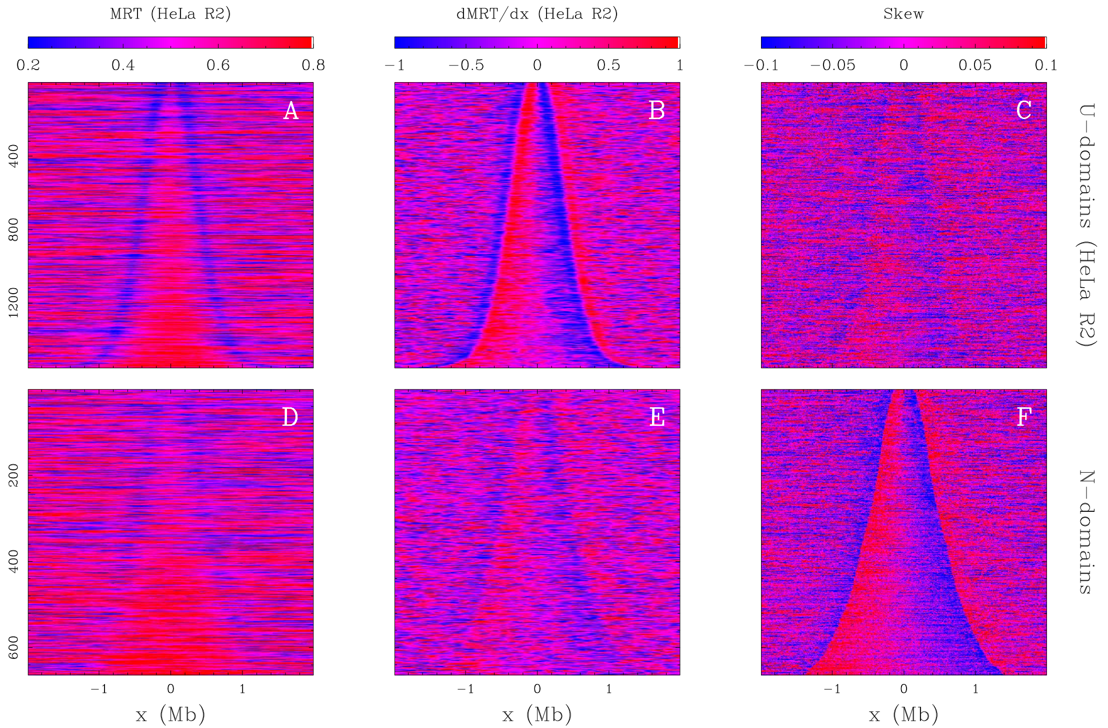

Figure S9. Same as in Supplementary Fig. S1 but for the HeLa cell line (Replicate experiment 2 : 1498 replication timing U-domains).
