# Supplementary material for: Replication Fork Polarity Gradients Revealed by Megabase-Sized U-Shaped Replication Timing Domains in Human Cell Lines
Source: PLoS Comput Biol. 2012 Apr 5;8(4):e1002443. doi: 10.1371/journal.pcbi.1002443 (PMC3320577; doi:10.1371/journal.pcbi.1002443)
Supplement: Figure S10 — Mean coverage (relative to the genome average) by DNase I hypersensitive zones, as a function of the distance to the closest U-domain border in H0287 (blue solid line : DNase GM06990, genome-wide mean value = 0.0107), in TL010 (blue dashed line : DNase GM06990, genome-wide mean value = 0.0107), in BJ R1 (light blue solid line : DNase BJtert, genome-wide mean value = 0.0164), in BJ R2 (light blue dashed line : DNase BJtert, genome-wide mean value = 0.0164), in HeLa R1 (magenta solid line : DNase HeLa S3, genome-wide mean value = 0.0136), in HeLa R2 (magenta dashed line : DNase HeLa S3, genome-wide mean value = 0.0136). (PDF) [file pcbi.1002443.s010.pdf]

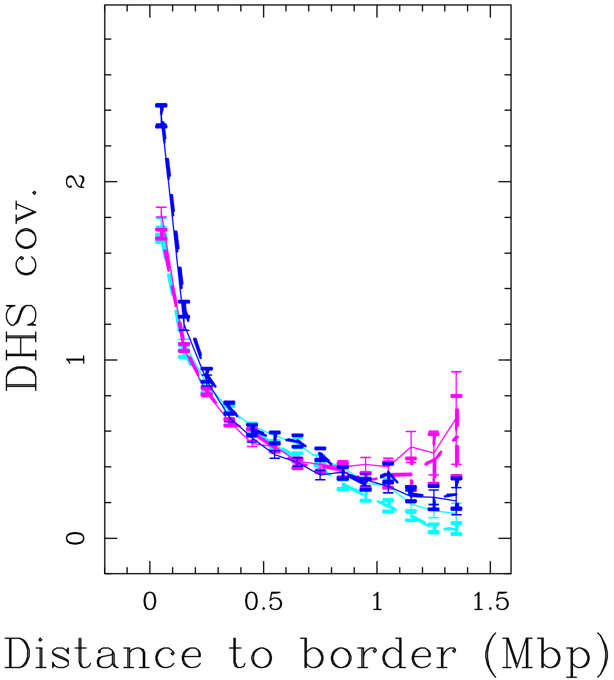

Figure S10. Mean coverage (relative to the genome average) by DNase I hypersensitive zones, as a function of the distance to the closest U-domain border in H0287 (blue solid line : DNase GM06990, genome-wide mean value = 0.0107), in TL010 (blue dashed line : DNase GM06990, genome-wide mean value = 0.0107), in BJ R1 (light blue solid line : DNase BJtert, genome-wide mean value = 0.0164), in BJ R2 (light blue dashed line : DNase BJtert, genome-wide mean value = 0.0164), in HeLa R1 (magenta solid line : DNase HeLa S3, genome-wide mean value = 0.0136), in HeLa R2 (magenta dashed line : DNase HeLa S3, genome-wide mean value = 0.0136).
