# Supplementary figures and images for: Replication Fork Polarity Gradients Revealed by Megabase-Sized U-Shaped Replication Timing Domains in Human Cell Lines
Source: PLoS Comput Biol. 2012 Apr 5;8(4):e1002443. doi: 10.1371/journal.pcbi.1002443 (PMC3320577; doi:10.1371/journal.pcbi.1002443)

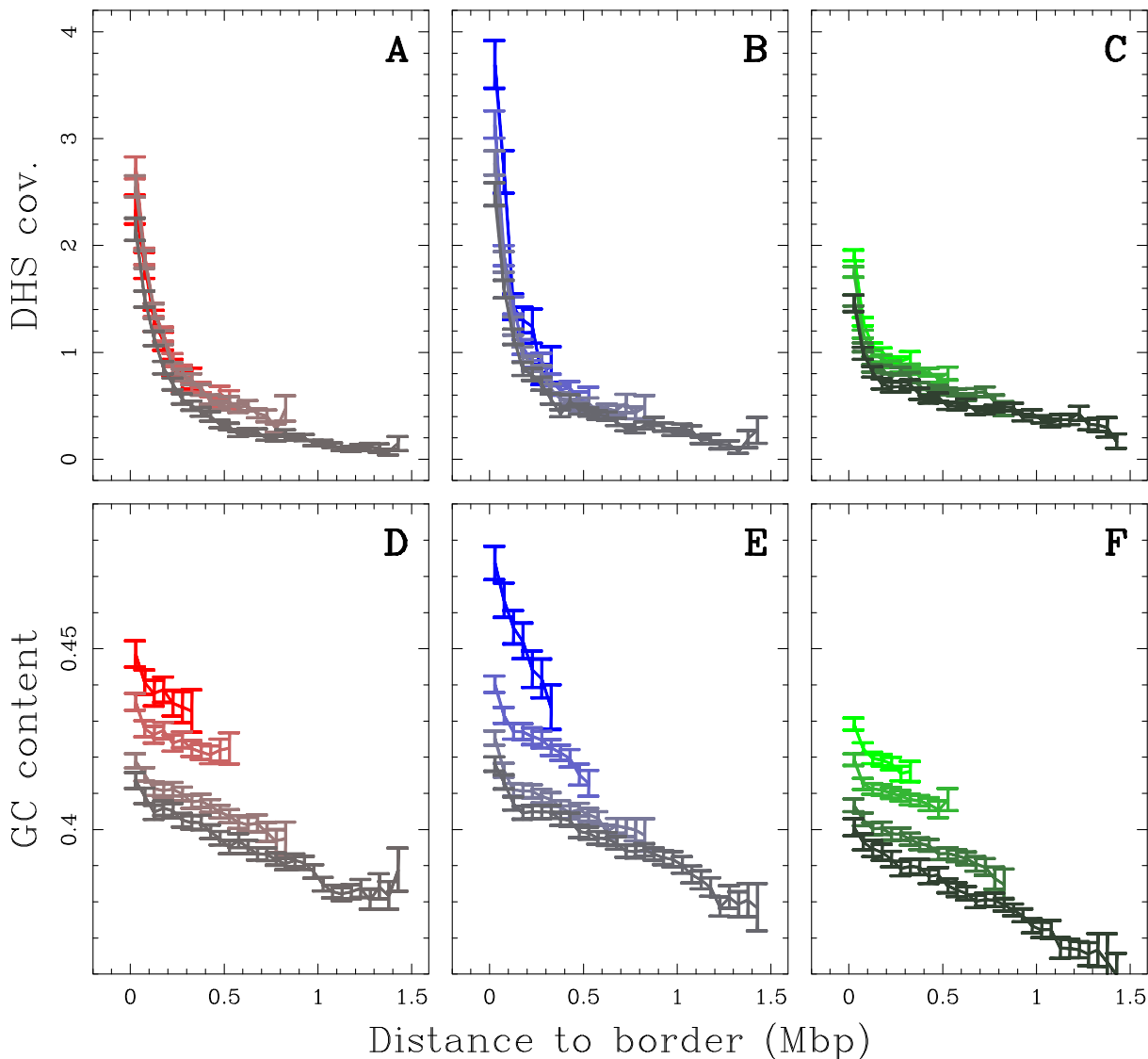

Supplement: Figure S11 — Mean coverage (relative to the genome average) of DNase I hypersensitive zones (A–C) and GC content (D–F) as a function of the distance to the closest U-domain border in K562 (A,D), GM06990 (B,E) and BG02 (C,F), for four U-domain size categories : L0.8 Mb, 0.8 MbL1.2 Mb, 1.2 MbL1.8 Mb and 1.8 MbL3 Mb from light to dark curves. (PDF) [file pcbi.1002443.s011.pdf]
