# Supplementary material for: Replication Fork Polarity Gradients Revealed by Megabase-Sized U-Shaped Replication Timing Domains in Human Cell Lines
Source: PLoS Comput Biol. 2012 Apr 5;8(4):e1002443. doi: 10.1371/journal.pcbi.1002443 (PMC3320577; doi:10.1371/journal.pcbi.1002443)
Supplement: Figure S12 — Same analysis as in Fig. 3 but restricted to replication timing U-domain borders that do not colocate within 100 kb with a N-domain border. (PDF) [file pcbi.1002443.s012.pdf]

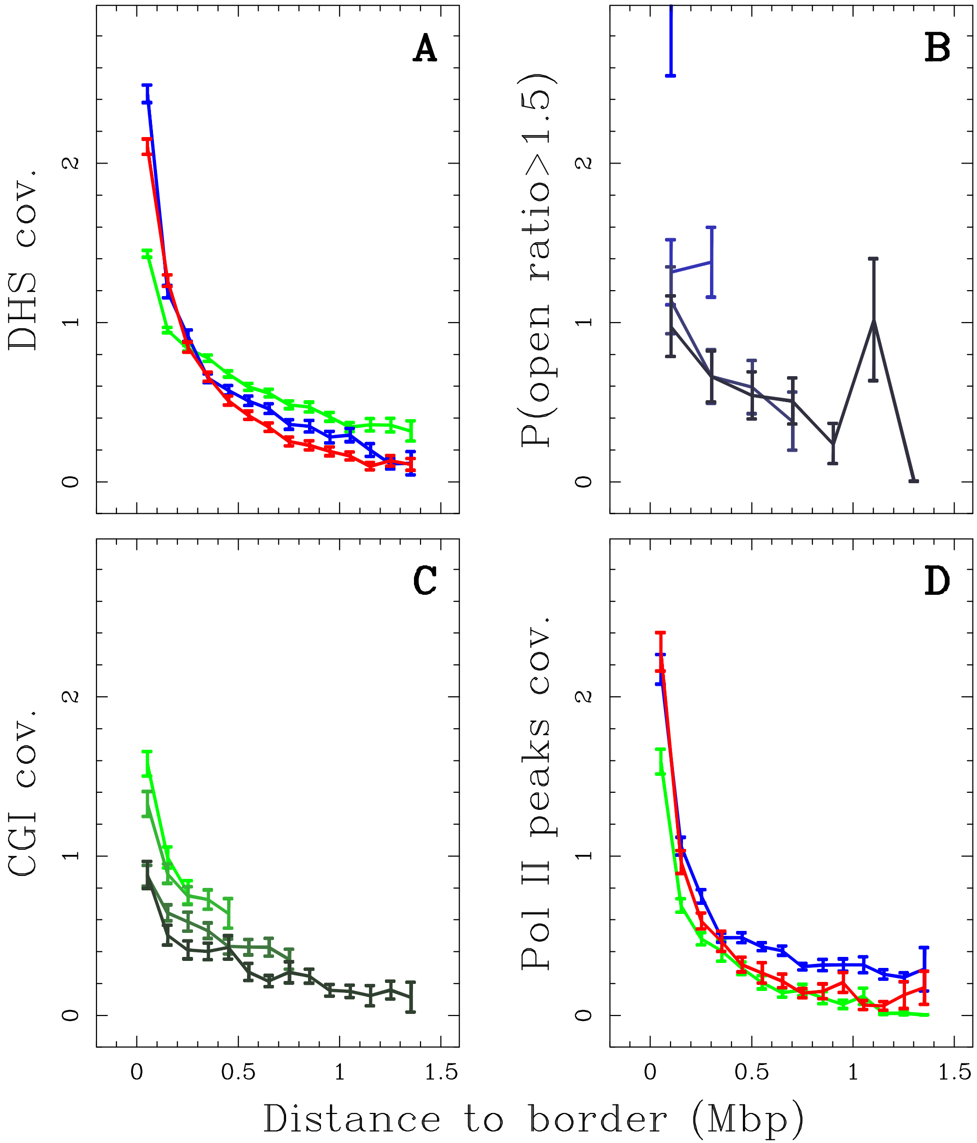

Figure S12. Same analysis as in Fig. 3 but restricted to replication timing U-domain borders that do not colocate within 100 kb with a N-domain border.
