# Supplementary material for: Replication Fork Polarity Gradients Revealed by Megabase-Sized U-Shaped Replication Timing Domains in Human Cell Lines
Source: PLoS Comput Biol. 2012 Apr 5;8(4):e1002443. doi: 10.1371/journal.pcbi.1002443 (PMC3320577; doi:10.1371/journal.pcbi.1002443)
Supplement: Figure S13 — (A) profile along a 23 Mb long fragment of human chromosome 5 that contains 5 detected skew N-domains (black horizontal bars). Each dot corresponds to the skew calculated for a window of 1 kb of repeat-masked sequence. The colors correspond to intergenic (black), genes (red) and genes (blue). (B) MRT profile from GM06990 cell line (blue curve); the vertical dashed blue lines correspond to the edges of 10 detected replication timing U-domains (horizontal blue bars). (C) Hi-C proximity matrix corresponding to intrachromosome interactions on the corresponding 23 Mb long fragment of human chromosome 5, as measured in the GM06990 cell line (Methods). Each pixel represents all interactions between a 100 kb locus and another 100 kb locus; intensity corresponding to the total number of reads is color coded according to the colormap (right). The dashed squares correspond to the 10 detected U-domains. (D) Number of interactions between two 100 kb loci versus the distance separating them (logarithmic scales) as computed genome wide (black) or in replication U-domains only, for four U-domain size categories : L0.8 Mb, 0.8 MbL1.2 Mb, 1.2 MbL1.8 Mb and 1.8 MbL3 Mb (from light to dark blue). (E) Ratio of the number of interactions between two 100 kb loci that are inside the same U-domain at equal distance from its center and the number of interactions between loci in different U-domains at equal distance from a U-domain border, versus the distance between them (logarithmic scales); the color coding is the same as in (D). The number of interactions per pair of 100 kb loci corresponds to averaging over the 882 U-domains detected in the GM06990 cell line (Table 2). (PDF) [file pcbi.1002443.s013.pdf]

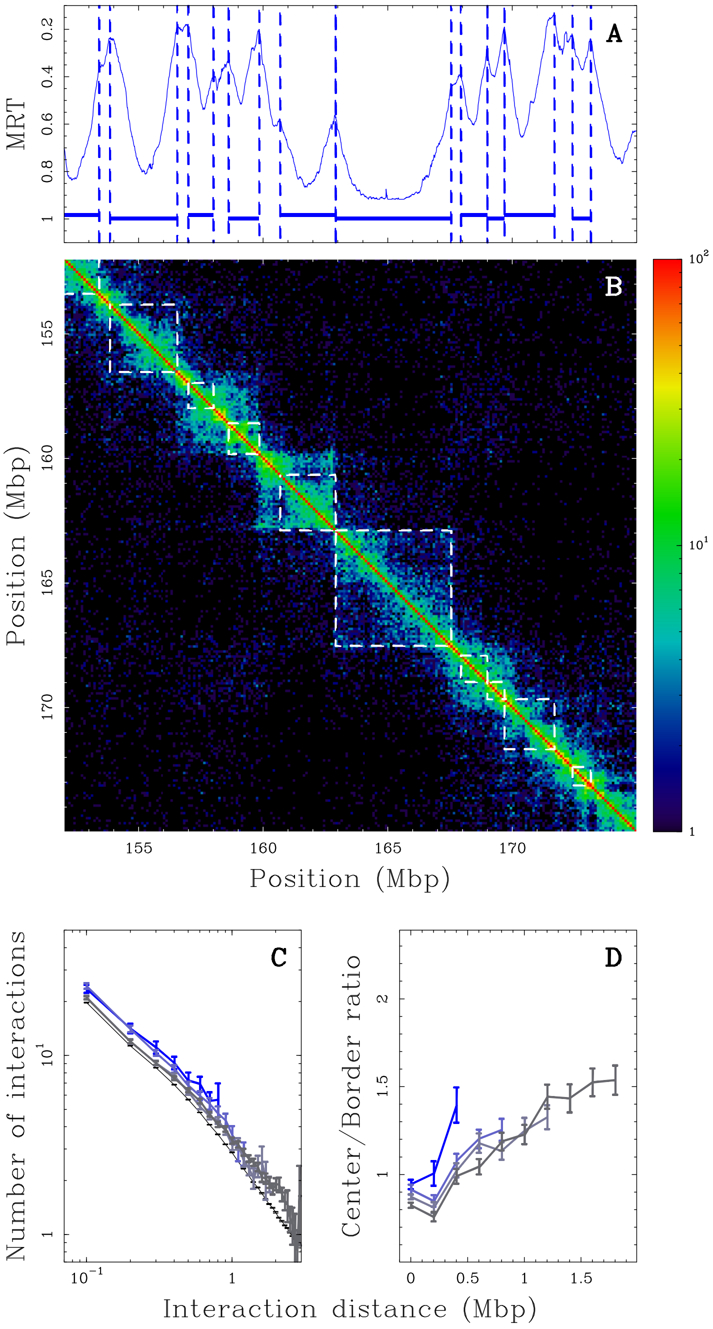

A
MRT
0.2
0.4
0.6
0.8
1
B
Position (Mbp)
155
160
165
170
Position (Mbp)
10^2
10^1
1
C
Number of interactions
10^1
1
10^{-1}
1
D
Center/Border ratio
2
1.5
1
0
0.5
1
1.5
Interaction distance (Mbp)

Figure S13. (A) $S = \frac{T-A}{T+A} + \frac{G-C}{G+C}$ profile along a 23 Mb long fragment of human chromosome 5 that contains 5 detected skew N-domains (black horizontal bars). Each dot corresponds to the skew calculated for a window of 1 kb of repeat-masked sequence. The colors correspond to intergenic (black), (+) genes (red) and (−) genes (blue). (B) MRT profile from GM06990 cell line (blue curve); the vertical dashed blue lines correspond to the edges of 10 detected replication timing U-domains (horizontal blue bars). (C) Hi-C proximity matrix corresponding to intrachromosome interactions on the corresponding 23 Mb long fragment of human chromosome 5, as measured in the GM06990 cell line (Methods). Each pixel represents all interactions between a 100 kb locus and another 100 kb locus; intensity corresponding to the total number of reads is color coded according to the colormap (right). The dashed squares correspond to the 10 detected U-domains. (D) Number of interactions between two 100 kb loci versus the distance separating them (logarithmic scales) as computed genome wide (black) or in replication U-domains only, for four U-domain size categories : L<0.8Mb, 0.8Mb<L<1.2Mb, 1.2Mb<L<1.8Mb and 1.8Mb<L<3Mb (from light to dark blue). (E) Ratio of the number of interactions between two 100 kb loci that are inside the same U-domain at equal distance from its center and the number of interactions between loci in different U-domains at equal distance from a U-domain border, versus the distance between them (logarithmic scales); the color coding is the same as in (D). The number of interactions per pair of 100 kb loci corresponds to averaging over the 882 U-domains detected in the GM06990 cell line (Table 2).
