# Supplementary material for: Replication Fork Polarity Gradients Revealed by Megabase-Sized U-Shaped Replication Timing Domains in Human Cell Lines
Source: PLoS Comput Biol. 2012 Apr 5;8(4):e1002443. doi: 10.1371/journal.pcbi.1002443 (PMC3320577; doi:10.1371/journal.pcbi.1002443)
Supplement: Figure S14 — (A) Normalized tag densities on a 25 Mb long fragment of chromosome 10, for the GM06990 cell line, and the corresponding computed MRT (white line). (B) “Denoised” normalized tag densities on the same genomic fragment and the corresponding MRT (white line). In (A) and (B) the tag densities for each S-phase fraction (G1–G2) are color coded using the color map situed at the top. (C) Comparison on the same genomic fragment of the MRT computed on the normalized tag densities (cyan line) and the MRT computed on the “denoised” normalized tag densities (blue line). (D) Probability density function (P.d.f.) of the genome-wide distribution of the normalized tag densities for each S-phase fraction from G1 to G2 from bottom to top (black histogram). The mode of the distribution is given by the red bar, the threshold used for denoising is given by the green bar. (PDF) [file pcbi.1002443.s014.pdf]

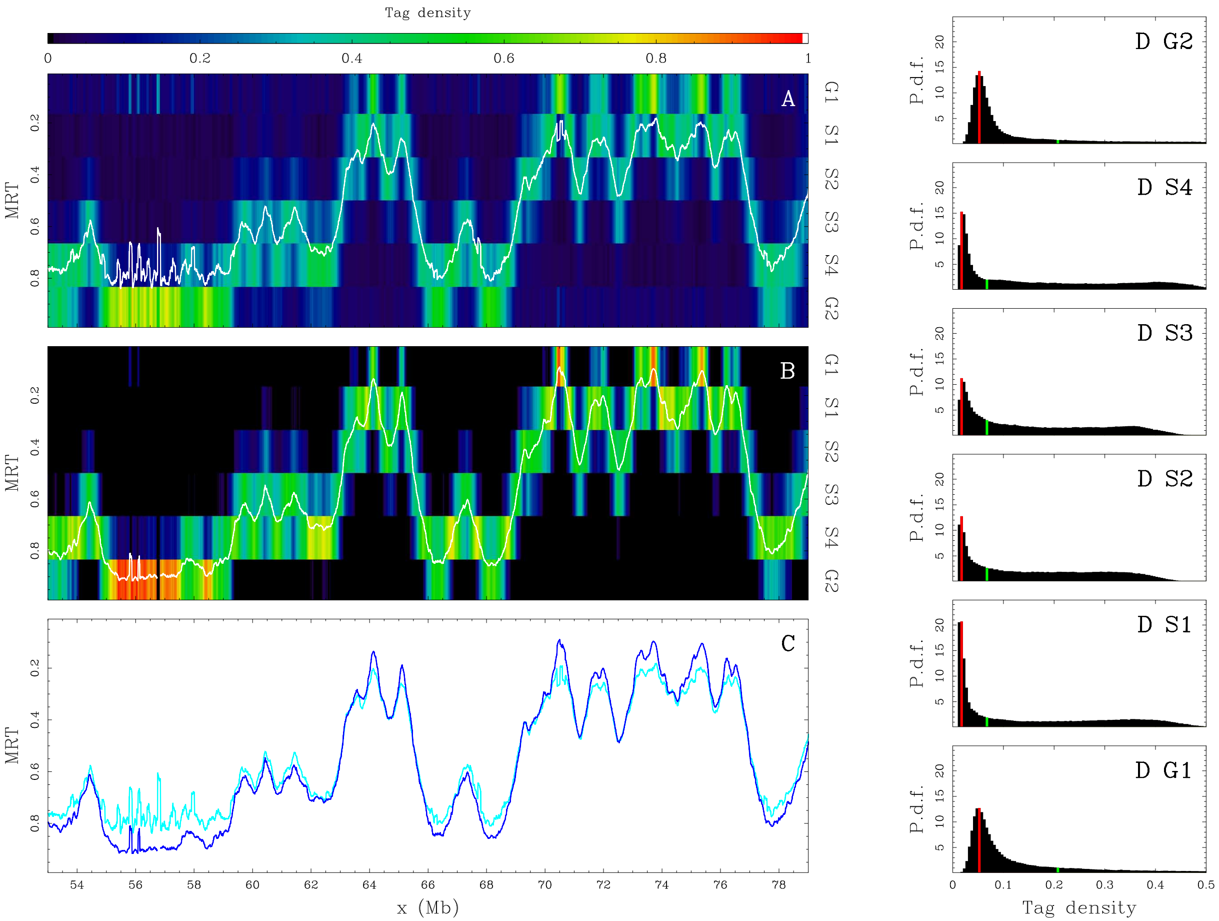

Figure S14. (A) Normalized tag densities on a 25 Mb long fragment of chromosome 10, for the GM06990 cell line, and the corresponding computed MRT (white line). (B) “Denoised” normalized tag densities on the same genomic fragment and the corresponding MRT (white line). In (A) and (B) the tag densities for each S-phase fraction (G1-G2) are color coded using the color map situed at the top. (C) Comparison on the same genomic fragment of the MRT computed on the normalized tag densities (cyan line) and the MRT computed on the “denoised” normalized tag densities (blue line). (D) Probability density function (P.d.f.) of the genome-wide distribution of the normalized tag densities for each S-phase fraction from G1 to G2 from bottom to top (black histogram). The mode m of the distribution is given by the red bar, the threshold $4m$ used for denoising is given by the green bar.
