# Supplementary material for: Replication Fork Polarity Gradients Revealed by Megabase-Sized U-Shaped Replication Timing Domains in Human Cell Lines
Source: PLoS Comput Biol. 2012 Apr 5;8(4):e1002443. doi: 10.1371/journal.pcbi.1002443 (PMC3320577; doi:10.1371/journal.pcbi.1002443)
Supplement: Figure S15 — (A) MRT profile obtained in K562 cell line along a 11.4 Mbp long segment of human chromosome 10. (B) Space-scale representation of second-order variations for the MRT profile presented in (A); (Equation (S7)) values are color coded using green (resp. orange) shades for negative (resp. positive) curvature (note that MRT axis is going downwards). Horizontal dashed line marks scale 300 kb used to detect regions of preferential replication initiation (vertical lines). Pairs of horizontal bars delineate the scale range where strong negative curvature is expected for parabolic U-shaped MRT profile. Regions delineated by two successive regions of preferential replication initiation are kept as U-domain if at their midpoint for some scale value in this range. (PDF) [file pcbi.1002443.s015.pdf]

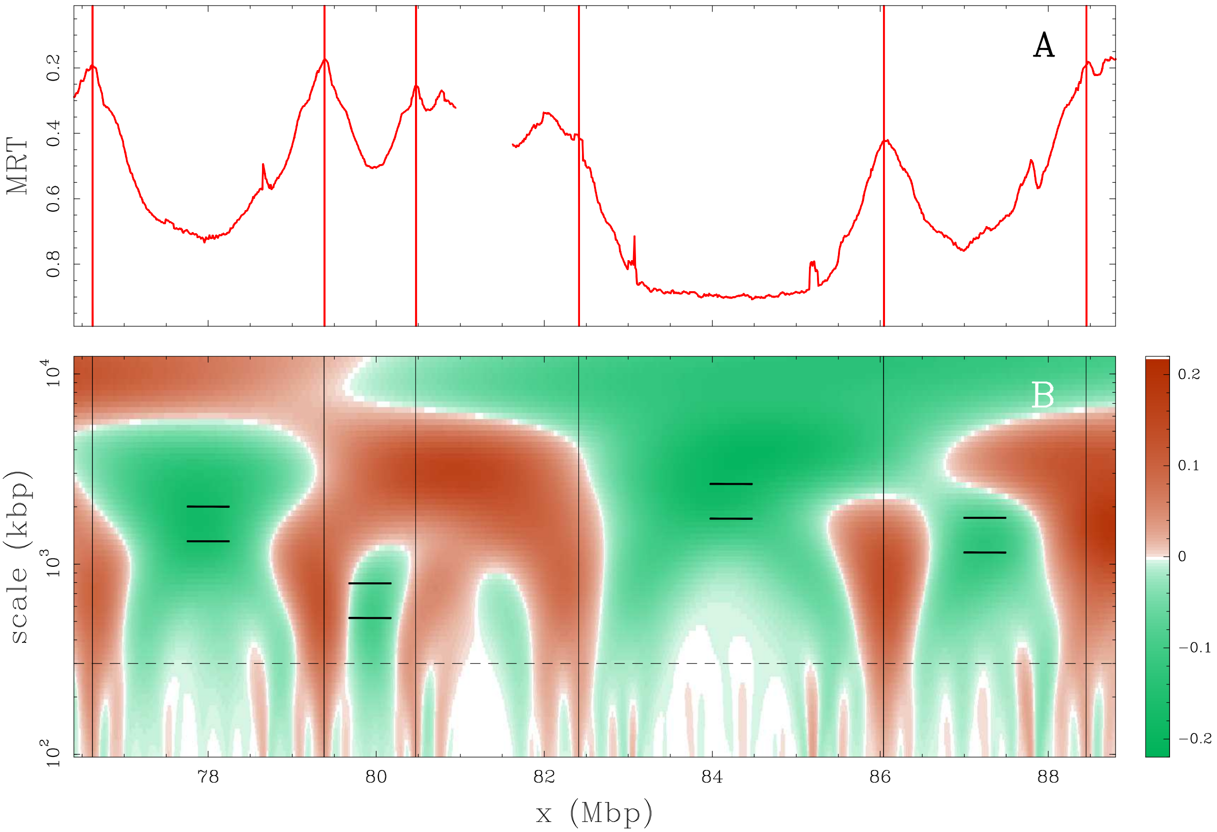

Figure S15. (A) MRT profile obtained in K562 cell line along a 11.4 Mbp long segment of human chromosome 10. (B) Space-scale representation of second-order variations for the MRT profile presented in (A); $T_{g^{(2)}}^{MRT}$ (Equation (S7)) values are color coded using green (resp. orange) shades for negative (resp. positive) curvature (note that MRT axis is going downwards). Horizontal dashed line marks scale 300 kb used to detect regions of preferential replication initiation (vertical lines). Pairs of horizontal bars delineate the scale range where strong negative curvature is expected for parabolic U-shaped MRT profile. Regions delineated by two successive regions of preferential replication initiation are kept as U-domain if $T_{g^{(2)}}^{MRT} \leq -0.04$ at their midpoint for some scale value in this range.
