# Supplementary material for: Replication Fork Polarity Gradients Revealed by Megabase-Sized U-Shaped Replication Timing Domains in Human Cell Lines
Source: PLoS Comput Biol. 2012 Apr 5;8(4):e1002443. doi: 10.1371/journal.pcbi.1002443 (PMC3320577; doi:10.1371/journal.pcbi.1002443)
Supplement: Table S1 — Pearson correlation (R values) of the derivative of MRT, dMRT/dx, between different pairs of human cell lines (Methods). dMRT/dx was calculated in non-overlapping 100 kb windows over the 22 human autosomes. All p-values are . (PDF) [file pcbi.1002443.s016.pdf]

R	BG02	K562	GM06990	H0287	TL010	BJ R1	BJ R2	HeLa R1	HeLa R2
BG02	1	0.42	0.39	0.39	0.35	0.39	0.41	0.36	0.31
K562	0.42	1	0.58	0.57	0.56	0.43	0.48	0.39	0.34
GM06990	0.39	0.58	1	0.9	0.84	0.47	0.5	0.41	0.34
H0287	0.39	0.57	0.9	1	0.84	0.47	0.49	0.41	0.35
TL010	0.35	0.56	0.84	0.84	1	0.45	0.45	0.37	0.32
BJ R1	0.39	0.43	0.47	0.47	0.45	1	0.92	0.52	0.48
BJ R2	0.41	0.48	0.5	0.49	0.45	0.92	1	0.53	0.48
HeLa R1	0.36	0.39	0.41	0.41	0.37	0.52	0.53	1	0.76
HeLa R2	0.31	0.34	0.34	0.35	0.32	0.48	0.48	0.76	1

Table S1. Pearson correlation (R values) of the derivative of MRT, dMRT/dx, between different pairs of human cell lines (Methods). dMRT/dx was calculated in non-overlapping 100 kb windows over the 22 human autosomes. All p-values are $< 10^{-16}$.
