# Supplementary material for: Replication Fork Polarity Gradients Revealed by Megabase-Sized U-Shaped Replication Timing Domains in Human Cell Lines
Source: PLoS Comput Biol. 2012 Apr 5;8(4):e1002443. doi: 10.1371/journal.pcbi.1002443 (PMC3320577; doi:10.1371/journal.pcbi.1002443)
Supplement: Table S2 — Number of matchings between replication timing U-domains in different pairs of cell lines including skew N-domains in the germline. A U-domain in a given cell line (column) was considered as matching a U-domain in another cell line (row) if more than 80% nucleotides of each of these U-domains were common to the two domains. (PDF) [file pcbi.1002443.s017.pdf]

| | Ndom | BG02 | K562 | GM06990 | H0287 | TL010 | BJ R1 | BJ R2 | HeLa R1 | HeLa R2 |
|---|---|---|---|---|---|---|---|---|---|---|
| Ndom | 663 | 157 | 119 | 119 | 109 | 86 | 83 | 100 | 120 | 124 |
| BG02 | 157 | 1534 | 197 | 189 | 170 | 119 | 207 | 246 | 238 | 228 |
| K562 | 119 | 197 | 876 | 251 | 239 | 205 | 184 | 191 | 197 | 188 |
| GM06990 | 119 | 189 | 251 | 882 | 536 | 373 | 184 | 196 | 173 | 181 |
| H0287 | 109 | 170 | 239 | 536 | 830 | 376 | 194 | 193 | 185 | 165 |
| TL010 | 86 | 119 | 205 | 373 | 376 | 664 | 141 | 148 | 131 | 138 |
| BJ R1 | 83 | 207 | 184 | 184 | 194 | 141 | 1150 | 855 | 334 | 306 |
| BJ R2 | 100 | 246 | 191 | 196 | 193 | 148 | 855 | 1247 | 356 | 332 |
| HeLa R1 | 120 | 238 | 197 | 173 | 185 | 131 | 334 | 356 | 1422 | 776 |
| HeLa R2 | 124 | 228 | 188 | 181 | 165 | 138 | 306 | 332 | 776 | 1498 |

**Table S2.** Number of matchings between replication timing U-domains in different pairs of cell lines including skew N-domains in the germline. A U-domain in a given cell line (column) was considered as matching a U-domain in another cell line (row) if more than 80% nucleotides of each of these U-domains were common to the two domains.
