# Supplementary material for: Replication Fork Polarity Gradients Revealed by Megabase-Sized U-Shaped Replication Timing Domains in Human Cell Lines
Source: PLoS Comput Biol. 2012 Apr 5;8(4):e1002443. doi: 10.1371/journal.pcbi.1002443 (PMC3320577; doi:10.1371/journal.pcbi.1002443)
Supplement: Table S3 — Number of matchings between randomly re-positioned replication timing U-domains in different pairs of cell lines including skew N-domains in the germline (1000 simulations were used to obtain the mean values). A U-domain in a given cell line (column) was considered as matching a U-domain in another cell line (row) if more than 80% nucleotides of each of these U-domains were common to the two domains. (PDF) [file pcbi.1002443.s018.pdf]

	Ndom	BG02	K562	GM06990	H0287	TL010	BJ R1	BJ R2	HeLa R1	HeLa R2
Ndom	13	30	21	22	21	18	25	26	27	26
BG02	30	72	45	46	42	35	58	63	66	66
K562	21	45	34	36	35	29	38	40	41	38
GM06990	22	46	36	40	39	32	39	41	41	38
H0287	21	42	35	39	37	31	37	38	39	35
TL010	18	35	29	32	31	27	31	32	32	29
BJ R1	25	58	38	39	37	31	49	52	53	52
BJ R2	26	63	40	41	38	32	52	56	56	57
HeLa R1	27	66	41	41	39	32	53	56	59	60
HeLa R2	26	66	38	38	35	29	52	57	60	62

Table S3. Number of matchings between randomly re-positioned replication timing U-domains in different pairs of cell lines including skew N-domains in the germline (1000 simulations were used to obtain the mean values). A U-domain in a given cell line (column) was considered as matching a U-domain in another cell line (row) if more than 80% nucleotides of each of these U-domains were common to the two domains.
