# Supplementary material for: Replication Fork Polarity Gradients Revealed by Megabase-Sized U-Shaped Replication Timing Domains in Human Cell Lines
Source: PLoS Comput Biol. 2012 Apr 5;8(4):e1002443. doi: 10.1371/journal.pcbi.1002443 (PMC3320577; doi:10.1371/journal.pcbi.1002443)
Supplement: Table S4 — Percentage of matchings between randomly re-positioned replication timing U-domains in different pairs of cell lines including skew N-domains in the germline (1000 simulations were used to obtain the mean values). A U-domain in a given cell line (column) was considered as matching a U-domain in another cell line (row) if more than 80% nucleotides of each of these U-domains were common to the two domains. (PDF) [file pcbi.1002443.s019.pdf]

	Ndom	BG02	K562	GM06990	H0287	TL010	BJ R1	BJ R2	HeLa R1	HeLa R2
Ndom	1.96	1.96	2.4	2.49	2.53	2.71	2.17	2.09	1.9	1.74
BG02	4.52	4.69	5.14	5.22	5.06	5.27	5.04	5.05	4.64	4.41
K562	3.17	2.93	3.88	4.08	4.22	4.37	3.3	3.21	2.88	2.54
GM06990	3.32	3	4.11	4.54	4.7	4.82	3.39	3.29	2.88	2.54
H0287	3.17	2.74	4	4.42	4.46	4.67	3.22	3.05	2.74	2.34
TL010	2.71	2.28	3.31	3.63	3.73	4.07	2.7	2.57	2.25	1.94
BJ R1	3.77	3.78	4.34	4.42	4.46	4.67	4.26	4.17	3.73	3.47
BJ R2	3.92	4.11	4.57	4.65	4.58	4.82	4.52	4.49	3.94	3.81
HeLa R1	4.07	4.3	4.68	4.65	4.7	4.82	4.61	4.49	4.15	4.01
HeLa R2	3.92	4.3	4.34	4.31	4.22	4.37	4.52	4.57	4.22	4.14

Table S4. Percentage of matchings between randomly re-positioned replication timing U-domains in different pairs of cell lines including skew N-domains in the germline (1000 simulations were used to obtain the mean values). A U-domain in a given cell line (column) was considered as matching a U-domain in another cell line (row) if more than 80% nucleotides of each of these U-domains were common to the two domains.
