# Supplementary material for: Replication Fork Polarity Gradients Revealed by Megabase-Sized U-Shaped Replication Timing Domains in Human Cell Lines
Source: PLoS Comput Biol. 2012 Apr 5;8(4):e1002443. doi: 10.1371/journal.pcbi.1002443 (PMC3320577; doi:10.1371/journal.pcbi.1002443)
Supplement: Text S1 — Supplementary methods: (i) Substitution rate matrix associated to replication (ii) Determination of mean replication timing profiles from experimental data and (iii) Detection of U-domains along mean replication timing profiles. (PDF) [file pcbi.1002443.s020.pdf]

Supplementary methods for:

Replication Fork Polarity Gradients Revealed by Megabase-sized U-shaped Replication Timing Domains in Human Cell Lines

Antoine Baker[1,2,3], Benjamin Audit[1,2,3], Chun-Long Chen[5], Benoit Moindrot[1,2], Antoine Leleu[1,2], Guillaume Guilbaud[6,†], Aurélien Rappailles[6], Cédric Vaillant[1,2,3], Arach Goldar[7], Fabien Mongelard[1,2,4], Yves d'Aubenton-Carafa[5], Olivier Hyrien[6], Claude Thermes[5], and Alain Arneodo[1,2,3,*]

1 Université de Lyon, F-69000 Lyon, France

2 Laboratoire Joliot-Curie, CNRS, Ecole Normale Supérieure de Lyon, F-69007 Lyon, France

3 Laboratoire de Physique, CNRS, Ecole Normale Supérieure de Lyon, F-69007 Lyon, France

4 Laboratoire de Biologie Moléculaire de la Cellule, CNRS, Ecole Normale Supérieure de Lyon, F-69007 Lyon, France

5 Centre de Génétique Moléculaire UPR 3404, CNRS, F-91198 Gif-sur-Yvette, France

6 Institut de Biologie de l'Ecole Normale Supérieure, CNRS UMR8197, Inserm U1024, 46 rue d'Ulm, 75005 Paris, France

7 Commissariat à l'énergie atomique, iBiTecS, 91191 Gif-sur-Yvette, France

† Present address: Medical Research Council Laboratory of Molecular Biology, Hills Road, Cambridge CB2 0QH, UK

∗ Email: alain.arneodo@ens-lyon.fr

Contents

Substitution rate matrix associated to replication **2**

Determining mean replication timing profiles from experimental data **4**

Detection of U-domains along mean replication timing profiles **5**

Substitution rate matrix associated to replication

We determined the nucleotide substitution pattern on each side (300 kb window) of the S-upward jumps [1]. Substitutions were tabulated in the human lineage since its divergence with chimpanzee using the macaque and orangutan as out-groups (Methods). Neutral substitution rates were reliably obtained by eliminating genic regions, and CpG islands which are unlikely to evolve neutrally. The substitution rate matrices so obtained in 300 kb windows immediately on the right (M_{right}) and on the left (M_{left}) of the putative replication origins provide estimate of the matrices $M_0 + M_R$ and $M_0 + \bar{M}_R$ associated to replication on the two strands. As reported just below M_{right} and $\bar{M}_{\mathrm{left}}$ provide consistent estimates of $M_R + M_0$:

$M_{\mathrm{right}} =$

	T	A	G	C
T	-0.4224	0.0602	0.1168	0.3622
A	0.0574	-0.4551	0.3540	0.1119
G	0.0783	0.3160	-0.5827	0.1208
C	0.2867	0.0789	0.1119	-0.5949

$\sim M_R + M_0$ (S1)

and

$\bar{M}_{\mathrm{left}} =$

	T	A	G	C
T	-0.4186	0.0597	0.1160	0.3634
A	0.0571	-0.4545	0.3530	0.1112
G	0.0772	0.3157	-0.5811	0.1196
C	0.2843	0.0791	0.1121	-0.5942

$\sim M_R + M_0$ (S2)

When decomposing these matrices into symmetric and antisymmetric parts, we get :

Symmetric parts

$M^s_{\text{right}} =$ (S3), $\sim M^s_R + M_0$

	T	A	G	C
T	-0.43875	0.05880	0.11435	0.35810
A	0.05880	-0.43875	0.35810	0.11435
G	0.07860	0.30135	-0.5888	0.11635
C	0.30135	0.07860	0.11635	-0.5888

and

$M^s_{\text{left}} =$ (S4), $\sim M^s_R + M_0$

	T	A	G	C
T	-0.43655	0.05840	0.11360	0.35820
A	0.05840	-0.43655	0.35820	0.11360
G	0.07815	0.30000	-0.58765	0.11585
C	0.30000	0.07815	0.11585	-0.58765

Antisymmetric parts

$M^a_{\text{right}} =$ (S5), $\sim M^a_R$

	T	A	G	C
T	0.01635	0.00140	0.00245	0.00410
A	-0.00140	-0.01635	-0.00410	-0.00245
G	-0.00030	0.01465	0.00610	0.00445
C	-0.01465	0.00030	-0.00445	-0.00610

and

$-M^a_{\text{left}} =$ (S6), $\sim M^a_R$

	T	A	G	C
T	0.01795	0.00130	0.00240	0.00520
A	-0.00130	-0.01795	-0.00520	-0.00240
G	-0.00095	0.01570	0.00655	0.00375
C	-0.01570	0.00095	-0.00375	-0.00655

We clearly see from these estimates that the condition $|M^a_R| \leq \epsilon |M^s_R + M_0|$ (Equation (8)) required to derive Equation (10) is satisfied.

Determining mean replication timing profiles from experimental data

We determined the mean replication timing profiles along the complete human genome using Repli-Seq data [2, 3]. This method consists in labeling newly synthesized DNA using a pulse of BrdU, sorting cells into several S-phase fractions using FACS and to reveal the locus of DNA synthesis in each fraction using anti-BrdU antibody combined to next-generation sequencing. For embryonic stem cells (BG02), three lymphoblastoid cell lines (GM06990, H0287, TL010) a fibroblast cell line (BJ, replicates R1 and R2) and erythroid K562 cell line, Repli-Seq tags for 6 FACS fractions were downloaded from the NCBI SRA website (Studies accession: SPR0013933) [2]. For a given cell line and for each S-phase fraction, we computed the tag densities in 100 kb windows, and following the authors [2] the tag densities were normalized to the same genome-wide sequence tag counts for each fraction, and a second normalization was performed so that at each genomic position, the sum over S-phase fractions be one. To filter out noise which could critically bias mean timing profile estimate (Supplementary Fig. S14A), we proceeded as follow. We noticed that the genome-wide distribution of the normalized tag density (Supplementary Fig. S14D) presents a mode at $0.01 < m < 0.08$ (mainly noise) and a long tail up to 1 (mainly corresponding to the replication signal). For each S-phase fraction we set to 0 the normalized tag density $< 4m$, and re-normalized at each genomic position by the sum over S-phase fractions. The mean replication timing profile computed on these denoised tag densities superimposes on the original one, but is much less noisy (Supplementary Fig. S14B,C).

For the HeLa cell line (replicates HeLa R1 and HeLa R2) the denoised tag densities were obtained from [3]. Instead of computing the S50 (median replication timing) as the authors in [3], we computed the mean replication timing (MRT).

Detection of U-domains along mean replication timing profiles

Within the approximation of constant fork velocity, the derivative of the MRT profile is related to the average fork polarity (Equation (2)). In the same manner, the replication timing profile for one replication cycle present positive curvature at replication origins and negative curvatures at termination sites (Fig. 6A) so that the second derivative of MRT profiles is related to the average initiation density minus the average termination density. Here, we propose to segment MRT at points of maximal curvature *i.e.* regions that present on average more initiation than termination events. This can be achieved using the continuous wavelet transform, which provides a powerful framework for the robust estimation of signal variations over any length scale [4, 5].

The wavelet-transform (WT) is a space-scale analysis which consists in expanding signals in terms of wavelets that are constructed from a single function, the analyzing wavelet, by means of dilations and translations [4,5]. When using the derivatives of the Gaussian function, namely $g^{(n)}(x) = \mathrm{d}^n g^{(0)}(x)/\mathrm{d}x^n$, with $g^{(0)}(x) = e^{-x^2/2}$, then the WT of MRT profile takes the following expression:

$$T^{MRT}_{g^{(n)}}(x,a) = \frac{1}{a}\int_{-\infty}^{+\infty} \langle t_{\mathcal{C}}(y)\rangle \; g^{(n)}\left(\frac{y-x}{a}\right) dy = (-a)^n \frac{\mathrm{d}^n}{\mathrm{d}x^n}\left(\frac{1}{a} g^{(0)}_a * \langle t_{\mathcal{C}}\rangle\right)(x) \;, \qquad \text{(S7)}$$

where x and a (> 0) are the space and scale parameters respectively. Equation (S7) shows that the WT computed with $g^{(n)}$ is proportional to the nth derivative of the MRT profile smoothed by a dilated version $g^{(0)}_a(x) = g^{(0)}(x/a)$ of the Gaussian function. This property is at the heart of various applications of the WT microscope as a very efficient multi-scale singularity tracking technique [4, 5].

In the space-scale representation of replication timing second-order variations provided by $T^{MRT}_{g^{(2)}}$, we delineated loci that present a local maximum in the MRT curvature profile at scale 300 kb (Supplementary Fig. S15; $T^{MRT}_{g^{(2)}} \geq 0.02$) as loci of preferential replication initiation. In a second step, we characterize the regions encompassed between two preferential replication initiation loci using the MRT curvature at their mid-point (Supplementary Fig. S15). We selected regions of length L with sufficiently negative values of $T^{MRT}_{g^{(2)}}$ (≤ -0.04; Equation (S7)) at some scale between $0.48L$ and $0.72L$ (for a parabolic shape profile of finite size L, the scale where extremal curvature is observed using the $T^{MRT}_{g^{(2)}}$ is proportional to L but also depends on the shape of the profile at the border of the region).

References

1. Chen CL, Duquenne L, Audit B, Guilbaud G, Rappailles A, et al. (2011) Replication-associated mutational asymmetry in the human genome. Mol Biol Evol 28: 2327–2337.

2. Hansen RS, Thomas S, Sandstrom R, Canfield TK, Thurman RE, et al. (2010) Sequencing newly replicated DNA reveals widespread plasticity in human replication timing. Proc Natl Acad Sci USA 107: 139–144.

3. Chen CL, Rappailles A, Duquenne L, Huvet M, Guilbaud G, et al. (2010) Impact of replication timing on non-CpG and CpG substitution rates in mammalian genomes. Genome Res 20: 447–457.

4. Mallat S (1998) A Wavelet Tour of Signal Processing. New York: Academic Press.

5. Arneodo A, Audit B, Decoster N, Muzy JF, Vaillant C (2002) Wavelet based multifractal formalism: Application to DNA sequences, satellite images of the cloud structure and stock market data. In: Bunde A, Kropp J, Schellnhuber HJ, editors, The Science of Disasters: Climate Disruptions, Heart Attacks, and Market Crashes, Berlin: Springer Verlag. pp. 26–102.
